# Profiling of epigenetic marker regions in murine ILCs under homeostatic and inflammatory conditions

**DOI:** 10.1084/jem.20210663

**Published:** 2022-08-08

**Authors:** Michael Beckstette, Chia-Wen Lu, Susanne Herppich, Elia C. Diem, Anna Ntalli, Aaron Ochel, Friederike Kruse, Beate Pietzsch, Katrin Neumann, Jochen Huehn, Stefan Floess, Matthias Lochner

**Affiliations:** 1 Department Experimental Immunology, Helmholtz Centre for Infection Research, Braunschweig, Germany; 2 Institute of Medical Microbiology and Hospital Epidemiology, Hannover Medical School, Hannover, Germany; 3 Bielefeld Institute for Bioinformatics Infrastructure, Department of Technology, Bielefeld University, Bielefeld, Germany; 4 Institute of Experimental Immunology and Hepatology, University Medical Center Hamburg-Eppendorf, Hamburg, Germany; 5 Institute of Infection Immunology, TWINCORE, Centre for Experimental and Clinical Infection Research; a joint venture between the Medical School Hannover and the Helmholtz Centre for Infection Research, Hannover, Germany

## Abstract

Epigenetic modifications such as DNA methylation play an essential role in imprinting specific transcriptional patterns in cells. We performed genome-wide DNA methylation profiling of murine lymph node–derived ILCs, which led to the identification of differentially methylated regions (DMRs) and the definition of epigenetic marker regions in ILCs. Marker regions were located in genes with a described function for ILCs, such as *Tbx21*, *Gata3*, or *Il23r*, but also in genes that have not been related to ILC biology. Methylation levels of the marker regions and expression of the associated genes were strongly correlated, indicating their functional relevance. Comparison with T helper cell methylomes revealed clear lineage differences, despite partial similarities in the methylation of specific ILC marker regions. IL-33–mediated challenge affected methylation of ILC2 epigenetic marker regions in the liver, while remaining relatively stable in the lung. In our study, we identified a set of epigenetic markers that can serve as a tool to study phenotypic and functional properties of ILCs.

## Teaser

In this study, the authors perform a genome-wide methylation analysis to define epigenetic signatures for murine ILCs and to assess similarities and differences to CD4^+^ T cells. Models of IL-33–mediated inflammatory challenge reveal tissue-specific epigenetic changes within ILC2 signature regions.

## Graphical Abstract

**Figure:**
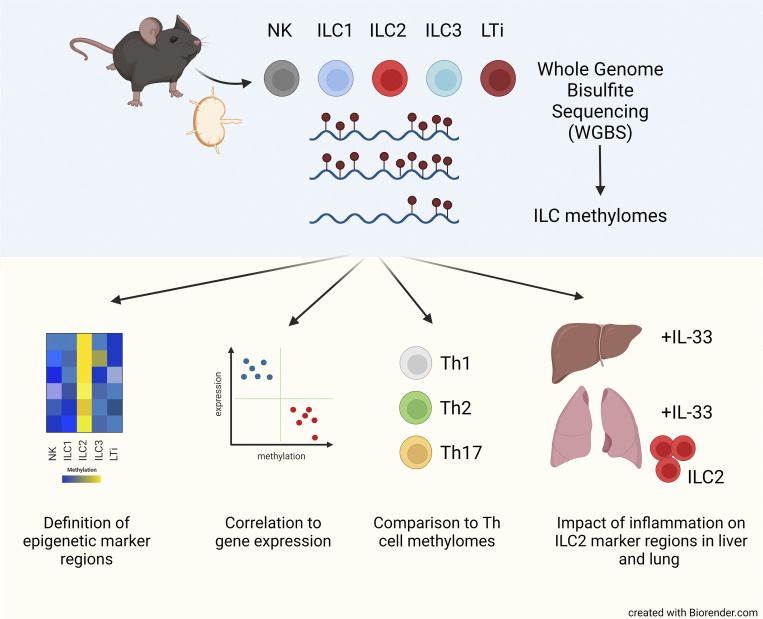

## Introduction

Innate lymphoid cells (ILCs) comprise an integral part of a complex and flexible immunological network with important roles under both homeostatic and inflammatory conditions. ILCs are currently classified into five different subsets: natural killer (NK) cells, ILC1, ILC2, ILC3, and lymphoid tissue inducer (LTi) cells, based on their ontogeny, the cytokines they produce, and the expression of transcription factors that are crucial for their maintenance and function (Vivier et al., 2018). Although ILCs lack antigen-specific activation and clonal selection, they share many phenotypical and functional properties with T cells. While NK cells show cytotoxic activity (therefore resembling CD8^+^ T cells), ILC1, ILC2, and ILC3 can be viewed as the innate counterparts of CD4^+^ T helper 1 (Th1), Th2, and Th17 lineages, respectively (Colonna, 2018). As such, ILC1 depend on the transcription factor T-bet and secrete the prototypical Th1 cytokine IFN-γ. ILC2 are defined by expression of Gata3 and their ability to secrete cytokines such as IL-5, IL-9, and IL-13. ILC3 and LTi cells are governed by RAR-related orphan receptor γt (RORγt) expression and produce the cytokines IL-17 and IL-22 (Colonna, 2018; Spits et al., 2013). Functionally, ILC1 and ILC3 contribute to immunity to viruses, bacteria, and fungi. ILC2s promote immunity to certain extracellular helminth parasites, and LTi cells are involved in the formation of secondary lymphoid organs during embryogenesis. Similar to CD4^+^ Th cells, however, ILCs may also contribute to tissue pathology under acute and chronic inflammatory conditions (Artis and Spits, 2015). Research published during recent years also revealed important roles for ILCs in various other physiological processes, including tissue homeostasis and remodeling, neuro-immune interaction, and tolerance (Branzk et al., 2018).

Transcriptomic analysis in conjunction with global epigenetic profiling is a powerful tool to gain novel insights into ILC lineage heterogeneity and function (Sciume et al., 2017). Recently, single-cell transcriptomes of human and murine ILCs confirmed the general classification into the ILC1-3 main lineages but suggested a significantly greater diversion of subpopulations, depending on the differentiation status, tissue location, and environmental stimuli such as the microbiota (Bjorklund et al., 2016; Gury-BenAri et al., 2016; Zeis et al., 2020). Epigenetic profiling of accessible chromatin regions and selective histone modifications revealed unique open chromatin landscapes in murine ILC subsets, fortifying the view of ILC1, ILC2, and ILC3 as distinct lineages (Gury-BenAri et al., 2016; Shih et al., 2016). Importantly, epigenetic marks appeared to be less sensitive to tissue localization and cell activation status than the transcriptome and therefore might reflect lineage relationship more reliably (Shih et al., 2016). Accessible chromatin and permissive histone marks were identified at the loci of lineage-specific transcription factors and signature cytokines (Gury-BenAri et al., 2016; Shih et al., 2016). However, while the expression of lineage signature cytokines was massively increased upon stimulation, the chromatin landscape remained relatively static, indicating that the corresponding genetic loci were epigenetically primed in ILC already before activation (Shih et al., 2016).

DNA methylation of CpG motifs represents a common epigenetic modification, which can act in concert with other epigenetic means to control the transcriptional program of immune cells (Zhang and Cao, 2019). In T cells, it has been demonstrated that specific CpG motifs located in the promoter and distal regulatory elements of the gene encoding for IFN-γ are hypomethylated in Th1 cells but hypermethylated in Th2 cells (Schoenborn et al., 2007). Moreover, demethylation of the *Ifng* promoter region was shown to be a prerequisite for IFN-γ expression and functional IFN-γ memory development (Dong et al., 2013; Winders et al., 2004). Likewise, the Th2-specific gene locus (*Il4*-*Il13*) is highly methylated in naive T cells and Th1 cell lines but demethylated in Th2 cells (Lee et al., 2002; Makar et al., 2003). Recently, specific demethylation within the promoters of *Il17a* and *Il17f*, as well as a unique signature of demethylated DNA regions, have been reported for Th17 cells (Thomas et al., 2012; Yang et al., 2015). A critical impact of DNA methylation has also been described for the development and maintenance of regulatory T (Treg) cells, as well as for their functional adaptation within peripheral tissues (Delacher et al., 2017; Floess et al., 2007; Ohkura et al., 2012). Interestingly, intense single-cell transcriptome studies suggest similar differentiation steps toward the establishment of tissue-specific ILC2 (Zeis et al., 2020). Together, these data highlight the importance of regulatory events for immune cell identity, stability, and function. However, a comprehensive analysis of rather stable regulatory mechanisms like changes in DNA methylation in ILC subsets has been lacking so far.

In this study, we established a genome-wide pattern of DNA methylation (the “methylome”) of murine ILC lineages. We identified a set of epigenetic marker regions for each ILC lineage in loci of genes with known as well as yet-unappreciated functions in ILCs and demonstrated a high correlation between the methylation status of the regions and the expression level of the associated genes. Although comparison to Th cell methylomes highlighted fundamental differences between ILCs and T cells, detailed analysis of the methylation level of ILC2 marker regions in Th2 cells revealed significant similarities. Moreover, the tissue environment seemed to influence both ILC2 residency and epigenetic adaption to environmental challenges such as inflammation. Therefore, our analysis suggests that changes in DNA methylation regulate differentiation into ILC lineages and critically respond to niche transition events.

## Results

### Identification of differentially methylated regions (DMRs) in ILCs

**Figure S1.**
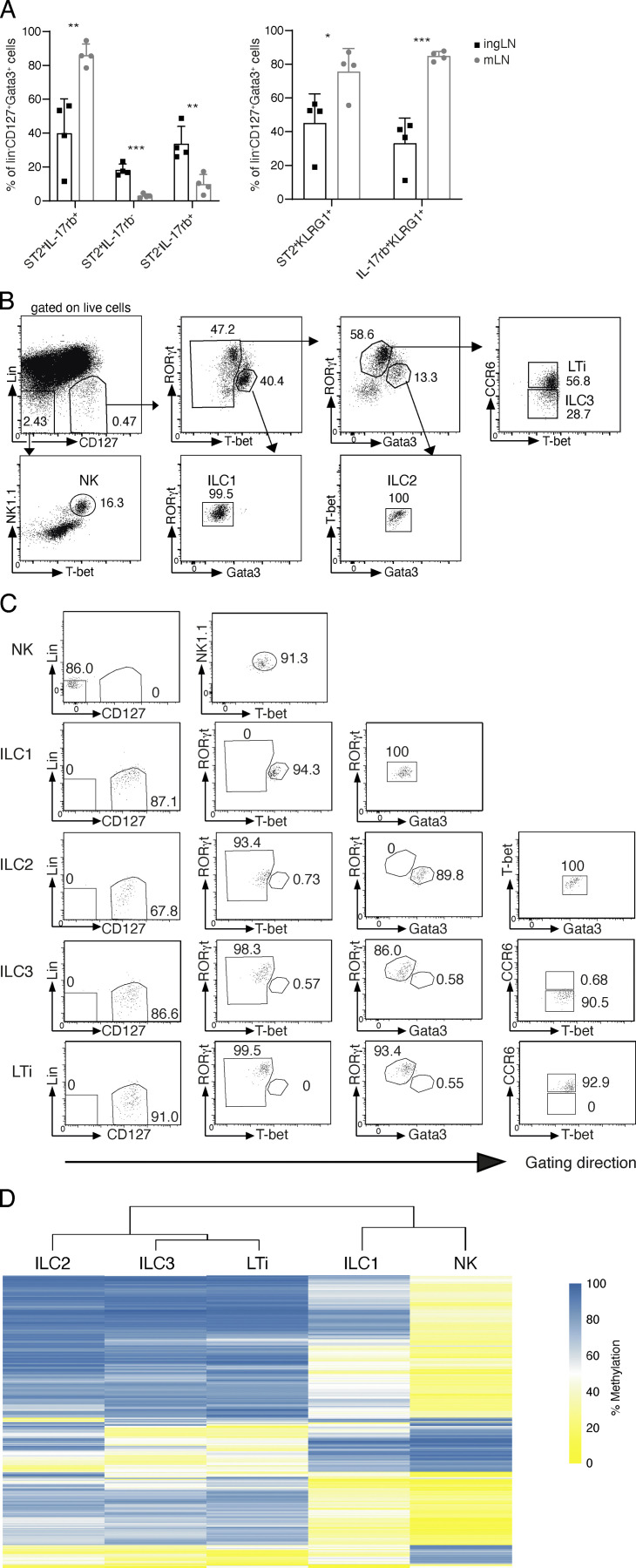
**Genome-wide methylation analysis of LN ILCs. (A)** Characterization of ILC2 in different LNs. Cells were isolated from inguinal (ingLN) or mesenteric (mLN) lymph nodes of Gata3 reporter mice (Gatir mice; Rao et al., 2020). ILC2 were gated as Lin^−^CD127^+^Gata3-YFP^high^ cells and further discriminated by expression of ST2, IL17RB, and Klrg1. Data pooled from *n* = 2 independent experiments with *n* = 2 mice per group. Statistical significance was analyzed using unpaired two-tailed Student’s *t* test with *, P ≤ 0.05; **, P ≤ 0.01; ***, P ≤ 0.001. **(B)** Sorting strategy for ILC WGBS. ILCs from pooled murine pLNs of WT C57BL/6J mice were sorted by indicated marker. Lineage markers (Lin) included CD3 and CD19. NK cells (Lin^−^CD127^−^NK1.1^+^Tbet^+^), ILC1 (Lin^−^CD127^+^Tbet^+^Gata3^−^RORgt^−^), ILC2 (Lin^−^CD127^+^Tbet^−^Gata3^+^RORgt^−^), ILC3 (Lin^−^CD127^+^CCR6^−^Tbet^−^Gata3^−^RORgt^+^), and LTi cells (Lin^−^CD127^+^CCR6^+^Tbet^−^Gata3^−^RORgt^+^). **(C)** Purity reanalysis of sorted ILCs. **(D)** Unsupervised hierarchical clustering of the top 1,000 DMRs among ILC subsets. The color represents the degree of mean methylation value, ranging from yellow (methylation level = 0) to blue (methylation level = 100%).

**Figure 1.**
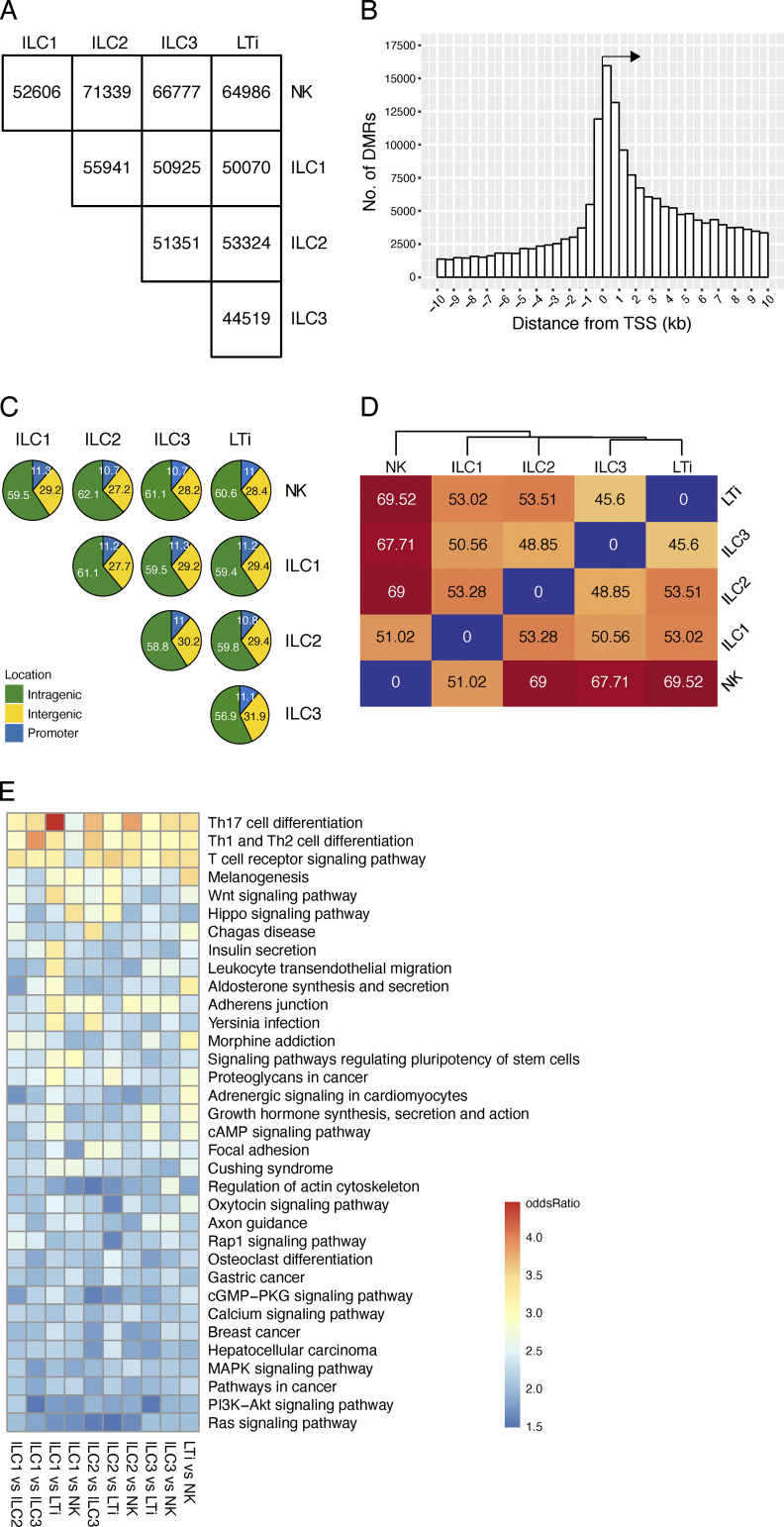
**Genome-wide methylation analysis of LN-derived ILCs.** For initial WGBS, ILC populations were pooled from >10 independent sorts of a total of *n* = 200 mice. WGBS was performed in unicates for NK cells, ILC1, ILC2, ILC3, and LTi cells. **(A)** Number of DMRs among ILC populations in pairwise comparisons. Methylomes were built from bisulfite sequencing data that were mapped against the reference genome. Numbers indicated the DMRs discovered by metilene software, containing at least three CpG motifs and a 25% methylation difference. **(B)** Number of discovered DMRs at various distances (x axis) relative to the TSS of the closest gene. **(C)** Pie charts indicating the location of the DMRs identified in groupwise comparisons. Numbers show the frequency of DMRs in intergenic, intragenic, or promoter regions according to their genomic position. **(D)** Euclidian sample distances of DMR methylation values from pairwise comparisons. Distance value and associated color code (red to blue) is shown. **(E)** Identification of pathways that are associated with identified DMRs by a KEGG-based pathway enrichment analysis. The odds ratio of the resulting pathways from the indicated pairwise comparisons were translated into a color code and ordered according to their value.

Whole-genome bisulfite sequencing (WGBS) was performed to allow the identification of specific DMRs in ILC lineages. Because ILCs are present only in very low numbers in murine tissues under homeostatic conditions, the isolation of a sufficient number of cells for WGBS represented a major challenge for this project. Therefore, we decided to purify ILCs from pooled peripheral lymph nodes (pLNs), where all of the ILC subpopulations could be found at reasonable frequencies. pLN-derived ILCs comprise a heterogeneous mixture of subpopulations; for examples ILC2 from inguinal and mesenteric LNs significantly differ in their expression of Klrg1 and the receptors for IL-25 and IL-33 (Fig. S1 A). To unambiguously identify the major ILC lineages, we fixed the cells isolated from the pLN and intracellularly stained them for the master transcription factors T-bet, Gata3, and RORγt. To reduce heterogeneity, we defined the ILC populations for cell sorting by the exclusive expression of one of the three master transcription factors. Performing several rounds of cell sorting, we were able to collect ≥2 × 10^5^ cells of the ILC lineages ILC1 (Lin^−^CD-127^+^RORγt^−^Gata3^−^T-bet^+^), ILC2 (Lin^−^CD127^+^RORγt^−^Gata3^+^-T-bet^−^), ILC3 (Lin^−^CD127^+^RORγt^+^-Gata3^−^T-bet^−^CCR6^−^), LTi cells (Lin^−^CD127^+^RORγt^+^Gata3^−^T-bet^−^CCR6^+^), and NK cells (Lin^−^CD127^−^NK1.1^+^T-bet^+^) in high purity (Fig. S1, B and C). A sequencing library for each ILC population was prepared from isolated genomic DNA and sequenced from both ends on an Illumina NovaSeq 6000 with a minimum of 2 × 10^8^ reads per single genome. The methylomes of the ILC populations were subsequently analyzed using the software metilene (Juhling et al., 2016), defining a DMR as a region with a minimum of three CpG motifs and ≥25% methylation difference in pairwise population comparisons. The analysis of the methylomes revealed the highest number of DMRs between NK cells and ILC2 (71,339), ILC3 (66,777), and LTi cells (64,986), whereas the lowest number (44,519) was identified between ILC3 and LTi cells (Fig. 1 A). Mouse reference genome–based annotation illustrated a high density of DMRs up- and downstream of transcription start sites (TSSs) of genes (Fig. 1 B). The majority of DMRs located to intragenic regions (∼60%), whereas only a smaller fraction was mapped to intergenic (∼30%) or promoter (∼10%) sites (Fig. 1 C). Euclidian distance analysis and hierarchical clustering of the top 1,000 unique DMRs showed that NK cells clustered distantly from ILC2, ILC3, and LTi cells, but not ILC1, and confirmed a close relationship between ILC3 and LTi cells (Figs. 1 D and S1 D). A global pathway analysis of the genes associated to the identified DMRs revealed a link to T cell–related processes such as Th1, Th2, and Th17 differentiation (Fig. 1 E), in line with the idea that ILCs and T cells share regulatory transcriptional pathways and are orchestrated by similar immune modules (Robinette and Colonna, 2016).

### Definition of epigenetic marker regions for ILCs

**Figure 2.**
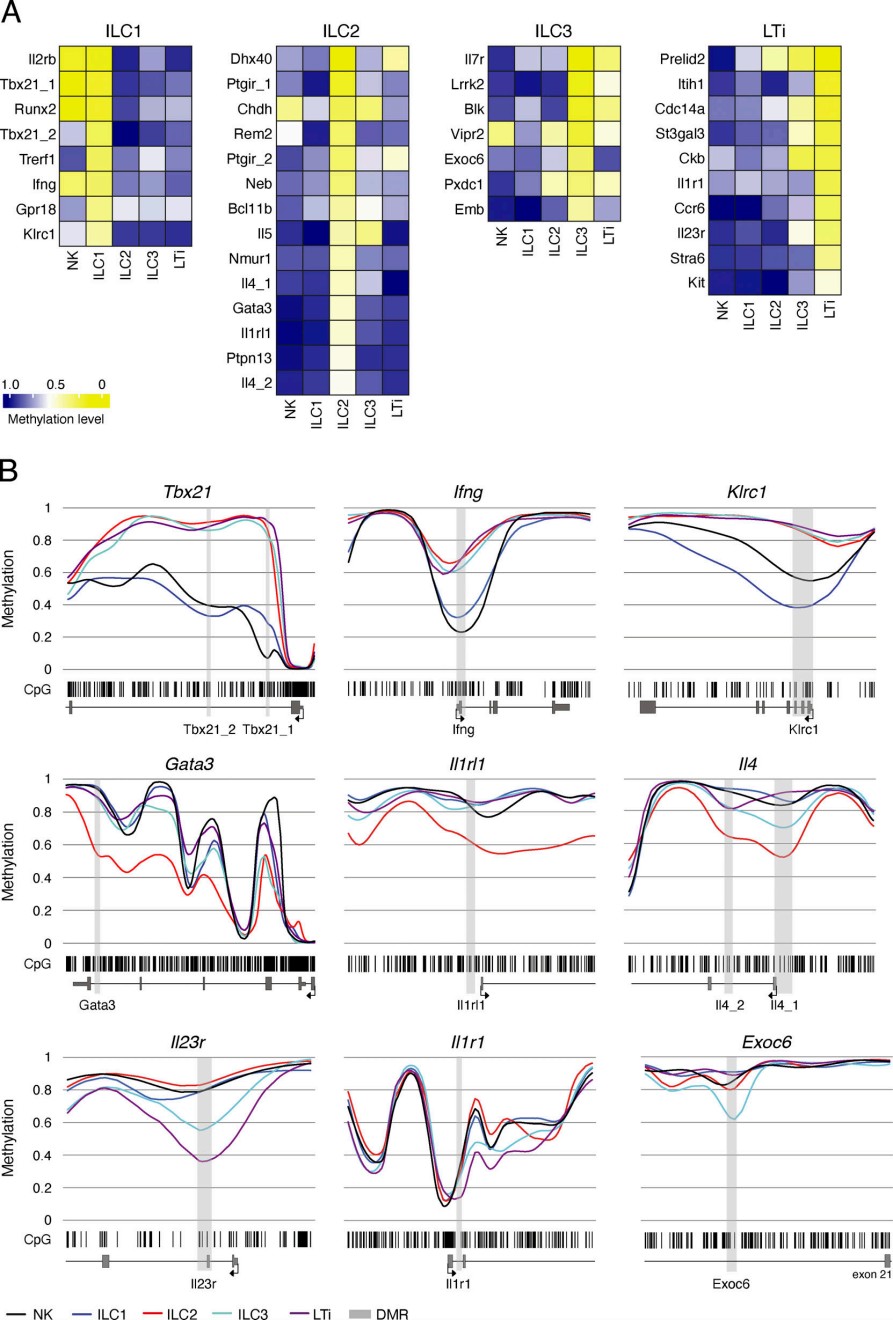
**Identification of epigenetic marker regions in LN-derived ILCs. (A)** Heatmaps showing the methylation level of selected epigenetic marker regions for ILC1, ILC2, ILC3, and LTi cells. Short DMRs produced by metilene software were extended to include adjacent differentially methylated CpG motifs to generate marker regions. The regions were named after the associated gene locus and numbered if more than one region was linked to a locus. The mean methylation value was calculated from the CpG motifs located within the marker region. The values were translated into a color code ranging from yellow (0% methylation = 0) via white (50% methylation = 0.5) to blue (100% methylation = 1.0). **(B)** Methylation profiles of marker-associated gene loci. Smoothed, linear display of CpG motifs (bar code), methylation values of the DMR (light gray box), and the surrounding gene body (exons in dark gray boxes, TSS indicated by arrow). Colored lines depict the methylation values ranging from 0 (0% methylation) to 1 (100% methylation) for each ILC subset (blue, ILC1; red, ILC2; cyan, ILC3; dark magenta, LTi; black, NK). Three selected gene loci for ILC1/NK (top), ILC2 (middle), and ILC3/LTi (bottom) are shown.

Among all identified DMRs, we next selected several DMRs for each ILC subset as potential candidates for ILC-specific epigenetic signatures. The basis for the selection of marker regions was the lists of DMRs derived from the pairwise comparisons, which were ranked from high to low methylation difference. The main selection criteria of the marker regions was a high degree (>40%) of differential methylation in the pairwise comparison. As the majority (>90%) of DMRs that were identified showed a lower difference in methylation (25–40%), this already excluded a large part of all DMRs. We also excluded DMRs that could not be clearly associated with an annotated gene. In addition, priority was given to DMRs containing higher numbers of differentially methylated CpGs and that showed high methylation difference across all pairwise comparisons and were thus demethylated exclusively in one ILC population. Based on these criteria, we selected 7–14 marker regions for each ILC population and named them after their associated genes (Fig. 2 A and Table S1 A). Of note, we did not completely focus on regions uniquely demethylated in one ILC population, but also included regions that showed a certain degree of demethylation also in other ILC populations. As depicted in the heat maps in Fig. 2 A, some marker regions in ILC1 and NK cells, or ILC3 and LTi cells, displayed a comparable degree of demethylation, indicating a substantial relationship among these ILC subsets. In contrast, the majority of the ILC2 markers seemed to be uniquely demethylated in this ILC subpopulation.

**Figure S2.**
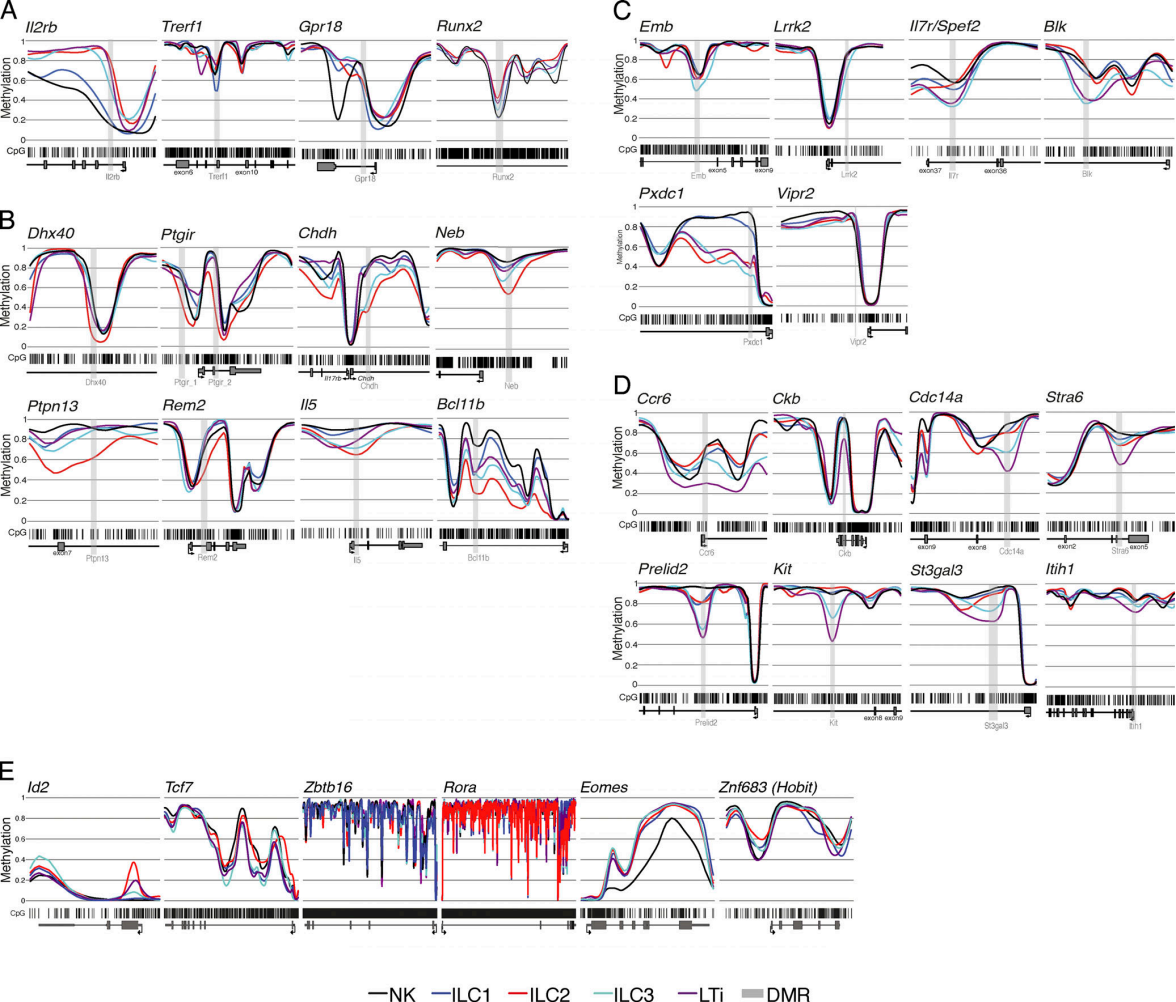
**Methylation profiles of CpG motifs within gene loci associated to ILCs. (A)** ILC1 marker regions (*Il2rb*, *Trerf1*, *Gpr18*, *Runx2*). **(B)** ILC2 marker regions (*Dhx40*, *Ptgir*, *Chdh*, *Neb*, *Ptpn13*, *Rem2*, *Il5*, *Bcl11b*). **(C)** ILC3 marker regions (*Emb*, *Lrrk2*, *Il7r*, *Blk*, *Pxdc1*, *Vipr2*). **(D)** LTi cell marker regions (*Ccr6*, *Ckb*, *Cdc14a*, *Stra6*, *Prelid2*, *Kit*, *St3gal3*, *Itih1*). **(E)** Methylation profiles covering the gene loci of *Id2*, *Tcf7*, *Zbtb16*, *Rora*, *Eomes*, and *Znf683*. Smoothed, linear display of CpG motifs (bar code) methylation values of the DMR (light gray box) and the surrounding gene body (exons in dark gray boxes, TSS indicated by arrow). Colored lines depict the methylation values ranging from 0 (0% methylation) to 1 (100% methylation) for each ILC subset (ILC1 = blue, ILC2 = red, ILC3 = cyan, LTi = dark magenta, NK = black).

To analyze the epigenetic landscape around selected marker regions, we generated methylation profiles that visualize the distribution of unmodified CpG motifs within the gene locus (Fig. 2 B and Fig. S2, A–D). For instance, clustered CpG motifs around the promoter region of the lineage transcription factor T-bet (encoded by *Tbx21*) were demethylated in all ILC subsets. However, differences in methylation levels became apparent in the first intron, where ILC1 and NK cells displayed higher levels of demethylation than other populations. A similar situation was observed at the *Gata3* locus, which displayed strong demethylation of CpG motifs at the promoter and the first two exons in all ILC populations, but a continuously demethylated pattern beyond intron 3 was exclusively found in ILC2 (Fig. 2 B). These findings indicate that although the promoter regions of both *Tbx21* and *Gata3* are demethylated in all ILCs, further epigenetic remodeling is necessary for stable transcription of the genes. Although NK cells and ILC1 shared most of the marker regions, the methylation profile of the *Klrc1* gene clearly revealed quantitative methylation differences across the whole locus, which is in accordance with higher protein expression on ILC1 (Krueger et al., 2017). The *Ifng* locus was demethylated in the promoter region and the first intron in both ILC1 and NK cells, and a similar pattern was observed for *Il4* in ILC2. Interestingly, an intronic region (intron 20) located at the end of the *Exoc6* gene locus showed a clear demethylation exclusively in ILC3. Specific demethylation of the promoter and its downstream region was observed for *Il23r* in ILC3 and LTi cells and *Il1rl1 *(coding for the IL-33 receptor) in ILC2. In contrast, the promoter region of *Il1r1* was demethylated in all ILC populations, while the first intron was exclusively demethylated in LTi cells with continued demethylation in the second intron for both ILC3 and LTi cells (Fig. 2 B). Of note, we did not find meaningful methylation differences in the loci of a number of genes with known importance for ILC differentiation or function such as *Id2*, *Tcf7*, and *Znf683* (Hobit), whereas the locus of *Eomes* displayed marked demethylation only in NK cells (Fig. S2 E). Within the huge gene loci of *Zbtb16* (*Plfz*) and *Rora*, several low-level hyper- and hypomethylated regions were found, but lacked a dominant modification (Fig. S2 E). A murine counterpart to the methylation-sensitive regulative region adjacent to the transcriptional start site of human *RORγt* was not found in ILCs (Schmidl et al., 2011). In addition, further methylation differences in the first intron were either not found (ILC1 vs. ILC3) or ≤30–35% and support the finding that murine RORγt expression is driven by histone modification (Bending et al., 2011). In summary, our WGBS approach enabled us to identify ILC-specific epigenetic marker regions and characterize the epigenetic landscape of associated genes. While some of the marker regions were associated with genes with known important functions in ILCs, our data also revealed epigenetic marks in genes that have not been linked to specific functions in ILCs so far, including several enzymes (*Chdh*, *Rem2*, *Ptpn13*, *Lrrk2*, *Ckb*), G protein–coupled receptors (*Gpr18*), transcription factors (*Trerf1*, *Runx2*), or receptors (*Stra6*; Fig. S2, A–D).

### DNA methylation pattern correlates with gene expression

**Figure S3.**
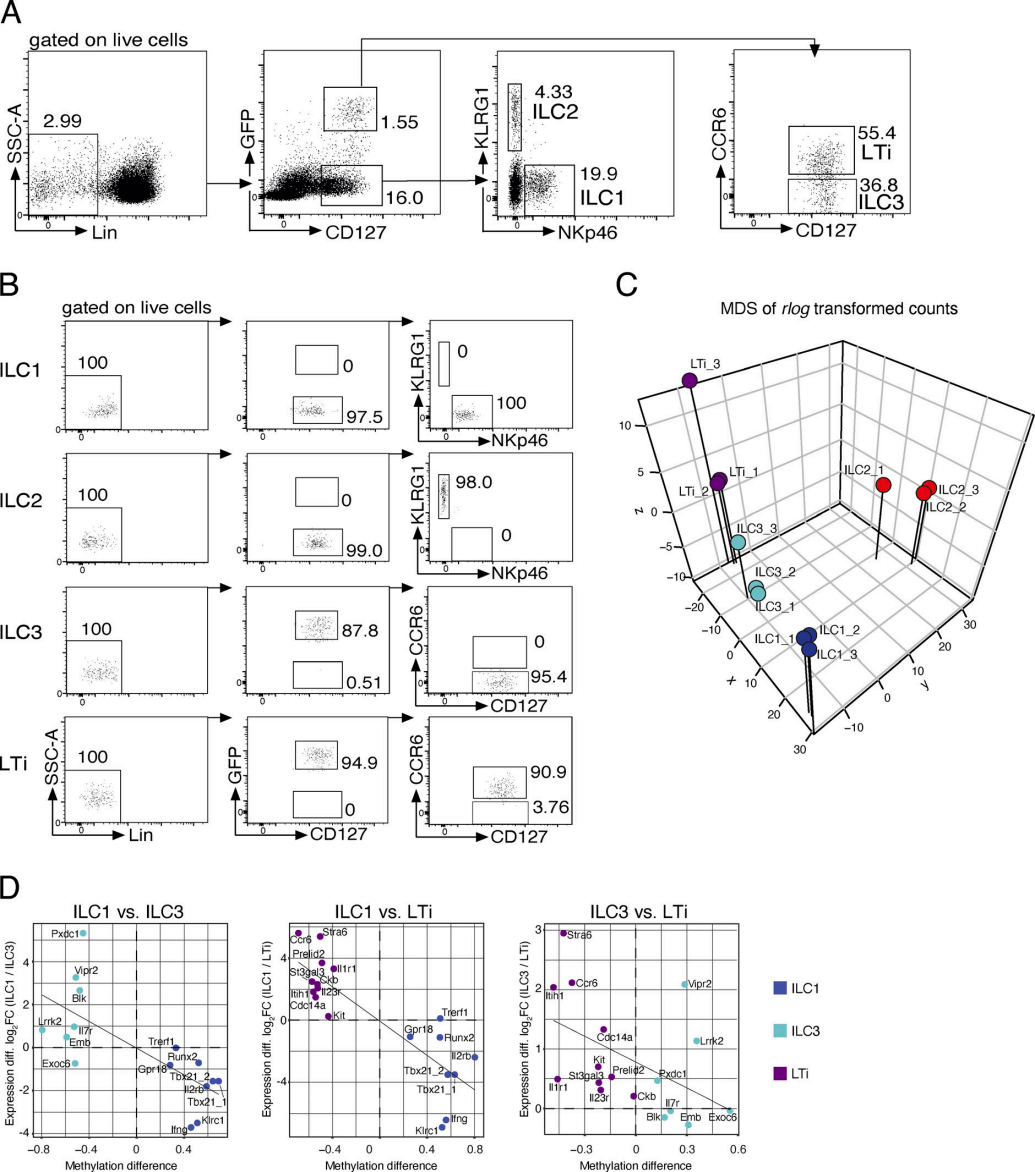
**RNA-seq of LN ILCs. (A)** Sorting strategy for ILC RNA-seq. ILCs were sorted from pooled pLNs of *Rorc*^GFP^ reporter mice by surface markers as ILC1 (Lin^−^CD127^+^RORγt^GFP−^NKp46^+^KLRG1^−^), ILC2 (Lin^−^CD127^+^RORγt^GFP−^NKp46^−^KLRG1^+^), ILC3 (Lin^−^CD127^+^RORγt^GFP+^CCR6^−^), LTi cells (Lin^−^CD127^+^RORγt^GFP+^CCR6^+^). SSC, side scatter. **(B)** Purity reanalysis of sorted ILCs. **(C)** Multidimensional scaling (MDS) of *rlog*-transformed expression counts in ILCs. Sample relationship similarity is shown in 3D plot including sample group color code (ILC1 = blue, ILC2 = red, ILC3 = cyan, LTi = dark magenta). **(D)** Correlation analyses visualize the relation between methylation status of marker regions (methylation difference, x axis) and associated gene expression (log2 fold-change, y axis). Analysis of ILC1 vs. ILC3, ILC1 vs. LTi, or ILC3 vs. LTi revealed *R* = −0.71 (P = 0.003), *R* = −0.84 (P < 0.001), or *R* = −0.49 (P = 0.047), respectively. Plots show the comparison between ILC1 (blue dots), ILC3 (cyan dots), or LTi (magenta dots) including the linear regression line.

**Figure 3.**
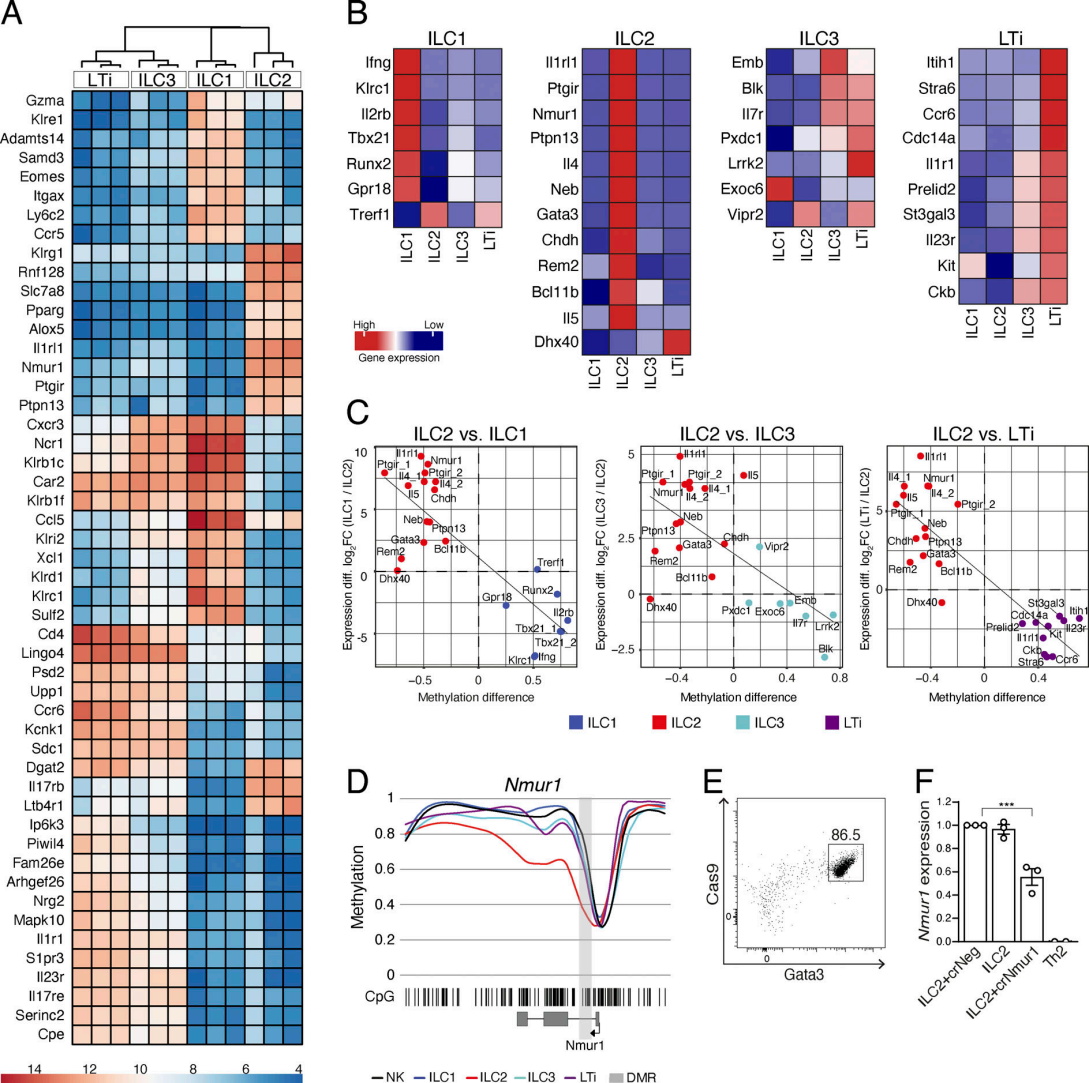
**Transcriptome analysis revealed high correlation between demethylation of marker regions and expression of the associated genes. (A)** Heatmap showing the top 50 most variable genes in an unbiased hierarchical clustering of ILC1-3 and LTi cells, as revealed by *DESeq2* expression analysis **(B)** Expression values (reads per kilobase maximum transcript length per million mapped reads) of marker-associated genes were translated into a heatmap and ranked according to the respective ILC population. **(C)** Correlation analyses visualize the relation between methylation status of marker regions (methylation difference, x axis) and associated gene expression (log_2_ fold-change [FC], y axis). Analysis of ILC1 vs. ILC2, ILC3 vs. ILC2, or LTi vs. ILC2 revealed *R* = −0.82 (P < 0.001), *R* = −0.67 (P < 0.001), or *R* = −0.86 (P < 0.001), respectively. Plots show the comparison between ILC2 (red dots) and ILC1 (blue dots), ILC3 (cyan dots), or LTi (magenta dots) including the linear regression line. **(D)** Methylation profile of the *Nmur1* locus for ILC1-3 and LTi cells, including smoothed linear display of CpG motifs (bar code), methylation values of the marker region (light gray box), and the surrounding gene body (exons in dark gray boxes, TSS indicated by arrow). **(E)** Representative flow cytometry plot showing Gata3 expression in ILC2 following 7 d of expansion in the presence of IL-2, IL-7, and IL-33. **(F)** sgRNA recognizing *Nmur1*-associated marker region (crNmur1) or negative control sgRNA (crNeg) were electroporated into in vitro–cultured ILC2 by electroporation. Nonelectroporated ILC2 and in vitro–differentiated Th2 cells served as additional controls. Graph depicts expression of Nmur1 determined 3 d after electroporation, shown as relative expression to Actb. RNA-seq was performed in *n* = 3 independent experiments and shown as triplicates (A) or mean values (B). Methylation data for the correlation analysis (C and D) was derived from the initial WGBS. In vitro targeting of ILC and Th2 cells (E and F) was performed in *n* = 3 independent experiments. Statistical significance was analyzed using unpaired two-tailed Student’s *t* test with ***, P ≤ 0.001.

In the next step, we aimed to correlate the methylation status of the marker regions with functional properties of the associated genes, in particular with the transcriptional activity. To do so, we performed RNA-based next-generation sequencing (RNA-seq) analysis of ILCs isolated from murine pLNs. As sorting ILC populations based on intracellular transcription factor staining was not possible due to the low RNA quality of the fixed cells, we used RORγt^GFP^ reporter mice and sorted cells according to surface marker and reporter gene expression in triplicates for ILC1, ILC2, ILC3, and LTi cell subpopulations (Fig. S3, A and B). Principal component analysis revealed that the gene expression patterns of ILC3 and LTi cells were more similar, while those of ILC1 and ILC2 were distinct (Fig. S3 C). This relationship was further confirmed by unsupervised hierarchical clustering of the top 50 differentially expressed genes, which grouped ILC3 and LTi cells closely together and left ILC1 and ILC2 as separated populations (Fig. 3 A). Notably, we observed that the list of the top 50 differentially expressed genes already contained some of the genes that are associated with our selected epigenetic marker regions, such as *Il1rl1*, *Nmur1*, *Ptgir*, *Ptpn13*, *Klrc1*, *Ccr6*, *Il1r1*, and *Il23r* (Fig. 3 A). Further analysis revealed that, except *Trerf1*, all of the genes that are associated with ILC1 epigenetic marker regions were also expressed by ILC1 (Fig. 3 B). Likewise, genes associated with ILC2 marker regions were both highly and specifically expressed by ILC2, with *Dhx40* being the only exception (Fig. 3 B). As expected, the genes that are associated with ILC3 and LTi marker regions were, in most cases, expressed to some extend by both populations (Fig. 3 B). Next, we directly correlated the methylation status of the marker regions with the expression levels of the corresponding genes, using ILC2 as a reference for the pairwise comparison to ILC1, ILC3, and LTi cells. As shown in our analysis (Fig. 3 C), we indeed observed a high correlation between levels of demethylation of the marker region and the expression of the corresponding gene for the majority of our selected marker regions. Notably, a high correlation was also observed for the comparison of gene expression and demethylation of ILC1 marker regions to ILC3 and LTi, whereas a similar comparison of ILC3 to LTi showed only a weak correlation (Fig. S3 D).

To specifically test the impact of a given marker region on the transcriptional activity of the locus, we targeted the epigenetic region in the *Nmur1* locus using CRISPR/Cas9 technology. Manipulation of the *Nmur1* locus served as a proof-of-principle approach, since our results showed that the *Nmur1* gene was highly and specifically expressed by ILC2 and contained a short pronounced demethylated region in the first intron (Fig. 3 D). To this end, ILC2 were isolated from the pLN of constitutively Cas9-expressing mice and expanded in vitro in the presence of IL-2, IL-7, and IL-33 (Fig. 3 E). After expansion, Cas9^+^Gata3^+^ ILC2s were electroporated with sgRNA recognizing the *Nmur1*-associated DMR. Quantitative PCR analysis revealed a reduction of *Nmur1* transcription to intermediate levels in targeted ILC2, compared with control ILC2 or Nmur1 low-expressing, in vitro differentiated Th2 cells. (Fig. 3 F). Thus, our findings confirm a strong correlation between gene expression and methylation status of the marker regions and support the assumption that these regions critically impact the transcriptional activity in ILCs.

### Epigenetic differences between ILCs and Th cells

**Figure S4.**
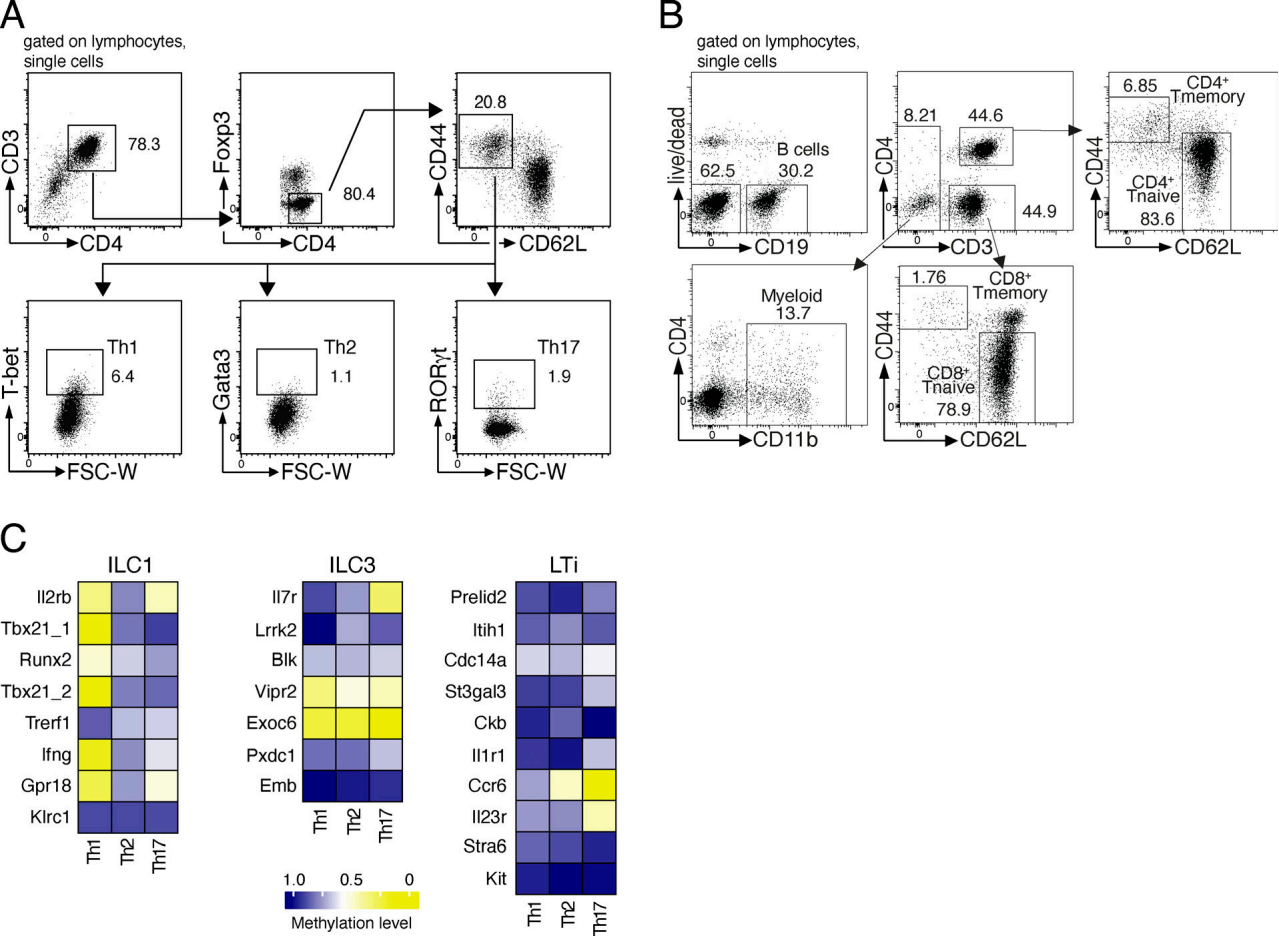
**Analysis of non-ILC immune cell subsets. (A)** Sorting strategy for the isolation of Th1, Th2, and Th17 cells. CD4^+^ cells from pooled pLNs and spleen of C57BL/6J mice were magnetically enriched by using anti-CD4 microbeads and the autoMacs Pro separator (Miltenyi Biotec). The enriched CD4^+^ cells were gated on single lymphocytes and sorted in high purity for Th1 (CD3^+^CD4^+^Foxp3^−^CD44^hi^CD62L^low^T-bet^+^), Th2 (CD3^+^CD4^+^Foxp3^−^CD44^hi^CD62L^low^Gata3^+^), and Th17 cells (CD3^+^CD4^+^Foxp3^−^CD44^hi^CD62L^low^RORγt^+^). **(B)** Sorting strategy for the isolation of main immune cell subsets. B cells (CD19^+^), myeloid cells (CD19^−^CD3^−^CD11b^+^), and both CD4^+^ and CD8^+^ T (CD19^−^CD3^+^) cells were sorted from murine lymph nodes. T cells were further distinguished into naive (CD44^−^CD62L^+^) and memory (CD44^+^CD62L^−^) populations. **(C)** Heatmaps showing the methylation level of ILC1, ILC3, and LTi epigenetic marker regions in Th1, Th2, and Th17 cells. The mean methylation value was calculated from the CpG motifs located within the marker region. The values were translated into a color code ranging from yellow (0% methylation = 0) via white (50% methylation = 0.5) to blue (100% methylation = 1.0).

**Figure 4.**
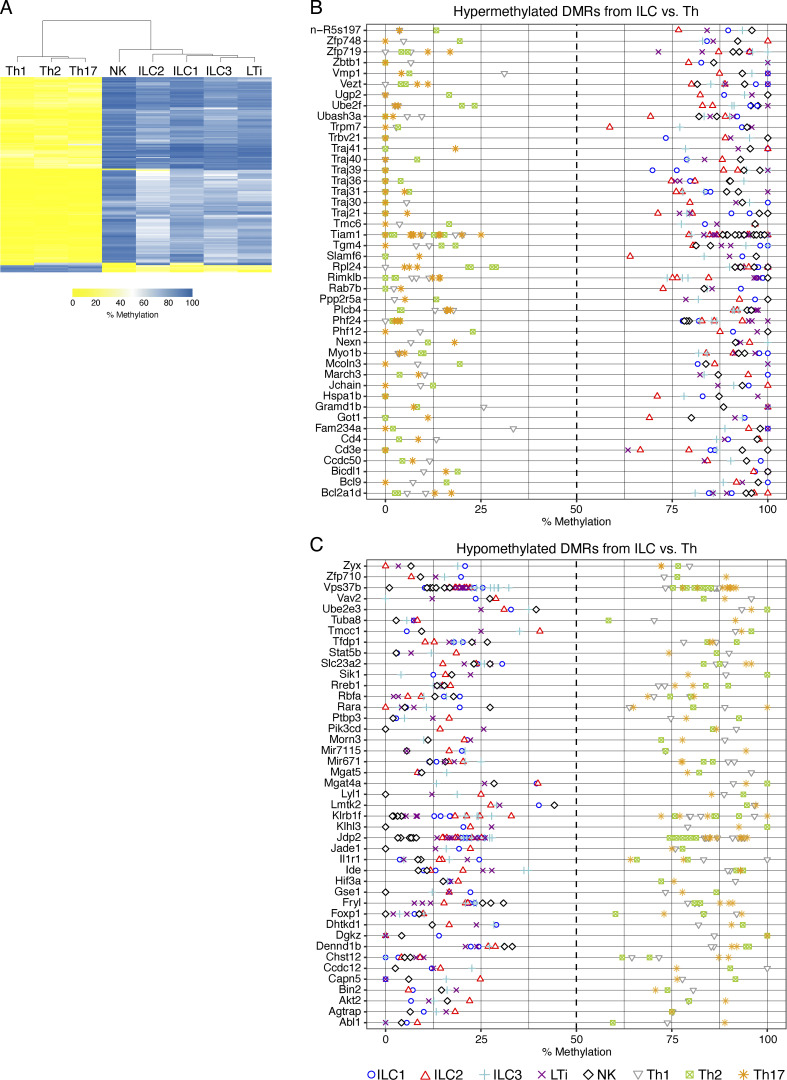
**Differences between the methylomes of ILCs and Th cells. (A)** Th1 (CD3^+^CD4^+^Foxp3^−^CD44^hi^CD62L^low^T-bet^+^), Th2 (CD3^+^CD4^+^Foxp3^−^CD44^hi^CD62L^low^Gata3^+^), and Th17 cells (CD3^+^CD4^+^Foxp3^−^CD44^hi^CD62L^low^RORγt^+^) were sorted by flow cytometry from pooled pLNs and spleen and subjected to WGBS. **(A)** Unsupervised hierarchical clustering of the top 1,000 DMRs among Th and ILC subsets. The color represents the degree of mean methylation value, ranging from yellow (methylation level = 0) to blue (methylation level = 100%). **(B and C)** Methylation values of the top 75 hypermethylated (B) and hypomethylated (C) DMRs derived from the comparisons of ILC vs. Th cell groups. The list is sorted according to the absolute mean methylation difference between the groups in descending order. Individual sample methylation values are represented by the displayed symbols. Multiple symbols indicate multiple DMRs in the same gene locus. Data for analysis of ILC methylomes was derived from the initial WGBS. Th cell populations were pooled from a total of *n* = 13 mice. WGBS was performed in unicates for Th1, Th2, and Th17 cells.

The initial DMR-based analysis revealed an overrepresentation of Th cell–specific developmental and receptor signaling pathways (Fig. 1 E). This is not unexpected, as it was reported before that ILC and Th cells share lineage transcription factors and effector cytokines. To shed more light on the relationship between these innate and adaptive immune cells, we generated methylomes from ex vivo–isolated Th1, Th2, and Th17 cells (Fig. S4 A). First, the most variable DMRs generated by pairwise comparisons among all ILCs and Th cells were subjected to hierarchical cluster analysis (Fig. 4 A and Table S2). Surprisingly, we did not detect a close relationship between cells driven by the same lineage transcription factor, such as Th1, ILC1, and NK cells, but a very clear separation between Th cells and ILCs. To better characterize the differences within the methylomes of ILC and Th cells, and to identify potential ILC- or Th cell–specific genetic regions, DMRs were further filtered for inversely methylated regions in the proximity of the promoter of annotated genes. As shown in Fig. 4 B, the top-ranked regions demethylated in Th cells contained DMRs associated with genes involved in T cell receptor assembly, such as T cell receptor α joining (*Traj*) genes and several genes linked to T cell receptor signaling, e.g., *Cd3*, *Cd4*, and *Ubash3a*, products of which build or interact with the TCR–CD3 complex and regulate its turnover (Ge et al., 2019), as well as the protein from *Slamf6*, which is required to augment T cell activation (Dragovich et al., 2019). Beside genes such as *Zbtb1* or *Bcl2a1d*, connected to T cell development, DMRs in metabolic genes such as *Got1*, which regulates Th17 and Th1 differentiation by glutaminase-dependent mechanisms (Johnson et al., 2018), were identified. DMRs that were uniquely demethylated in ILCs were identified in the gene loci of transcription factors Stat5b and Lyl1, both expressed during ILC development (Liu et al., 2021; Villarino et al., 2017), or *Rara*, which is highly expressed by ILCs (Kim et al., 2015). In addition to several genes with immunological functions, such as *Klrb1f* or *Foxp1*, we surprisingly also identified ILC-specific DMRs in genes such as *Dennd1b*, *Akt2*, *Abl*, or *Dgkz*, playing a role in different immune cells, including T cells (Martin et al., 2012; Singh et al., 2019; Tanaka et al., 2021; Yang et al., 2016b; Zipfel et al., 2004). In summary, uniquely demethylated regions in Th cells are located in genes involved in T cell metabolism, development, and T cell receptor signaling, whereas uniquely demethylated regions in ILCs appear in genes of transcription factors and signaling mediators expressed in diverse immune cells.

### ILC2 signatures overlap partially with Th2 cells and carry T cell–regulating factor binding sites in overrepresented motifs

**Figure 5.**
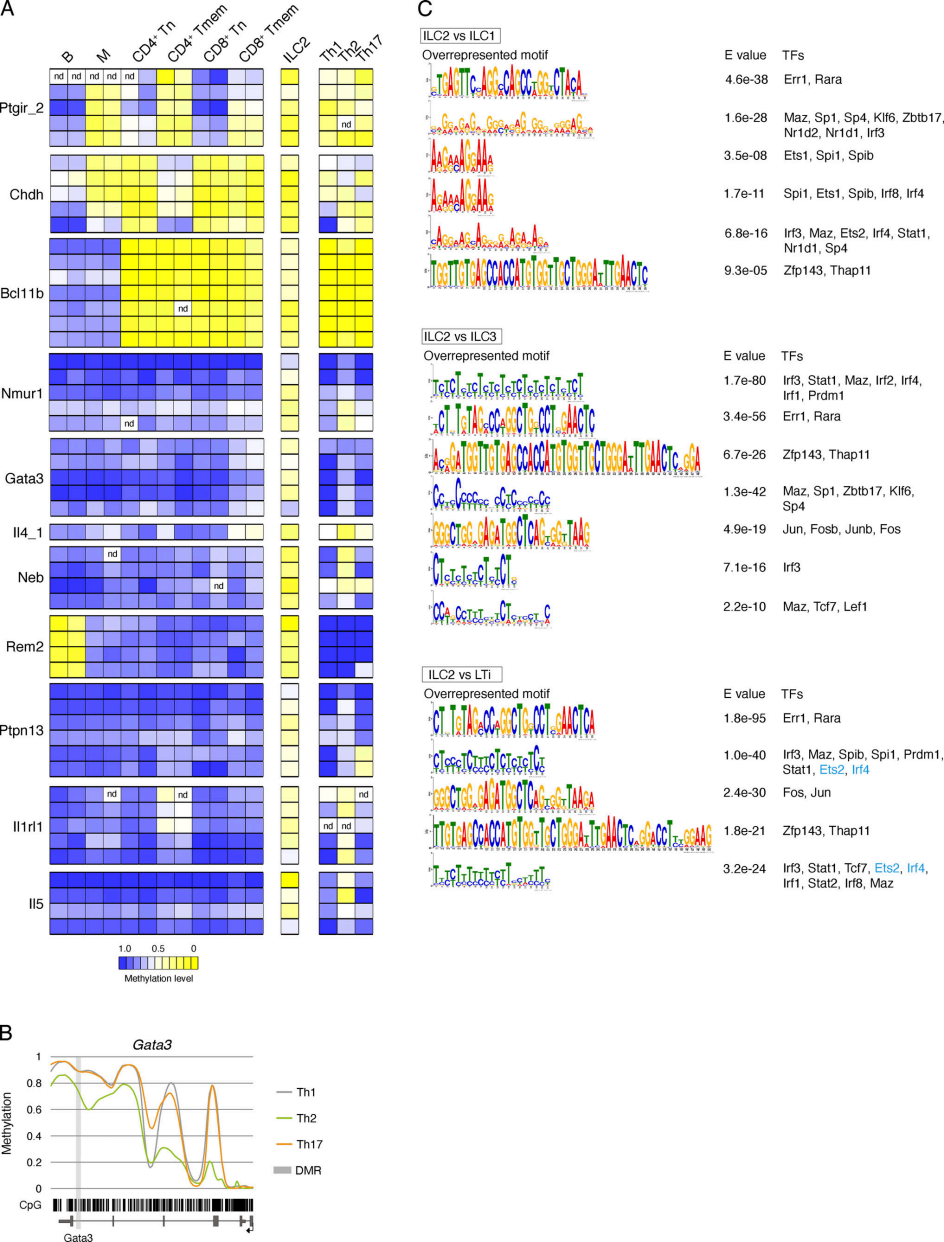
**ILC2 show partial signature overlaps with Th cells and carry T cell–regulating factor binding sites in overrepresented motifs. (A)** B cells (CD19^+^), myeloid cells (CD19^−^CD3^−^CD11b^+^), and naive (CD44^−^CD62L^+^) and memory (CD44^+^CD62L^−^) CD4^+^ and CD8^+^ (CD19^−^CD3^+^) T cells were sorted by flow cytometry from the spleen of WT mice and analyzed for the methylation of CpG motifs within ILC2 marker regions by pyrosequencing. The mean methylation value of all CpG motifs within each region was calculated and transformed into a color-coded box, ranging from blue (100% methylation) and white (50% methylation) to yellow (0% methylation). Each box represents the methylation value of one CpG motif. The white box labeled with *nd* represents an invalid sequencing signal. The experiment was performed in *n* = 2 independent sorts. Methylation values shown for the CpG motifs of ILC2 were extracted from the WGBS data of Th1, Th2, and Th17 cells and LN-derived ILC2. B, B cells; M, myeloid cells; Tn, naive T cells; Tmem, memory T cells. **(B)** Methylation profile of *Gata3* gene locus. Smoothed, linear display of CpG motif (bar code) methylation values of the DMR (light gray box) and the surrounding gene body (exons in dark gray boxes, TSS indicated by arrow). Colored lines depict the methylation values ranging from 0 (0% methylation) to 1 (100% methylation) for each Th subset (Th1 = gray, Th2 = green, Th17 = orange). **(C)** Overrepresented sequences and corresponding E values of identified overrepresented motifs (MEME analysis) among DMRs of ILC2 comparisons are shown as indicated. Motifs containing transcription factors (TFs) not expressed in ILC2 (RNA-seq analysis) or motifs without any transcription factor binding site were excluded. Transcription factors in blue are differentially expressed in ILC2.

For a more detailed analysis, we set the focus on the ILC2 marker regions described above, since the majority of these regions displayed pronounced and specific demethylation only in this ILC population. As demethylation of the marker regions may also occur in non-ILC immune cell populations, we first performed pyrosequencing within the ILC2 marker regions to determine the methylation status in selected main immune cell subsets. For this purpose, B cells (CD19^+^), myeloid cells (CD19^−^CD3^−^CD11b^+^), and naive (CD3^+^CD62L^+^CD44^−^) as well as memory (CD3^+^CD62L^−^CD44^+^) subsets of both CD4^+^ T and CD8^+^ T cells, were sorted from splenocytes of healthy mice (Fig. S4 B). As expected, some of the marker regions were significantly demethylated also in other immune cell subsets, such as *Bcl11b* in all T cells, *Rem2* in B cells, *Ptgir* in T memory and myeloid cells, and *Chdh* in most of the analyzed cell types (Fig. 5 A and Table S3).

Analysis of the ILC2 marker regions in Th cell subsets Th1, Th2, and Th17 revealed comparable levels of demethylation in *Ptgir*, *Bcl11b*, and also *Chdh*, which still displayed stronger demethylation in ILC2 (Fig. 5 A). While all other ILC2 marker regions remained largely methylated in Th1 and Th17 cells, similar levels of demethylation between ILC2 and Th2 cells were found in *Il4*, *Il5*, and *Il1rl1*, whereas the epigenetic regions in *Nmur1*, *Gata3*, and *Neb* were more strongly demethylated in ILC2. The regions in *Rem2* and *Ptpn13* showed no or only weak demethylation in Th2 cells. Thus, our analysis revealed a partial methylation overlap between ILC2 and Th2 cells, and similar results were obtained for ILC1 regions in Th1 or ILC3/LTi regions in Th17 cells (Fig. S4 C). A detailed inspection of the *Gata3* locus revealed demethylated regions within Th2 that do not appear in Th1 or Th17 cells (Fig. 5 B). However, the locus showed also a different profile in ILC2 with a more distinct demethylation in the last intron (Fig. 2 B). Interestingly, a motif overrepresentation analysis performed for ILC2 vs. ILC1, ILC3 and LTi DMRs, followed by a search for transcription factor binding sites, resulted in factors known to play a role in T cells, like family members of T cell factor/lymphoid enhancer factor (TCF/LEF), IFN regulatory factor (IRF), POK/ZBTB, STAT, JUN, and FOS (Evans and Jenner, 2013; Kang and Malhotra, 2015; Fig. 5 C). Together, these data indicate that despite expected pathway and regulation similarities between ILC2 and Th2 cells, specific methylation differences still exist. Single marker regions may therefore not be exclusively demethylated in ILC2, but the overall signature of marker regions might be specific for ILC2.

### Impact of inflammatory stimuli on the methylation of ILC2 marker regions in liver and lung

**Figure S5.**
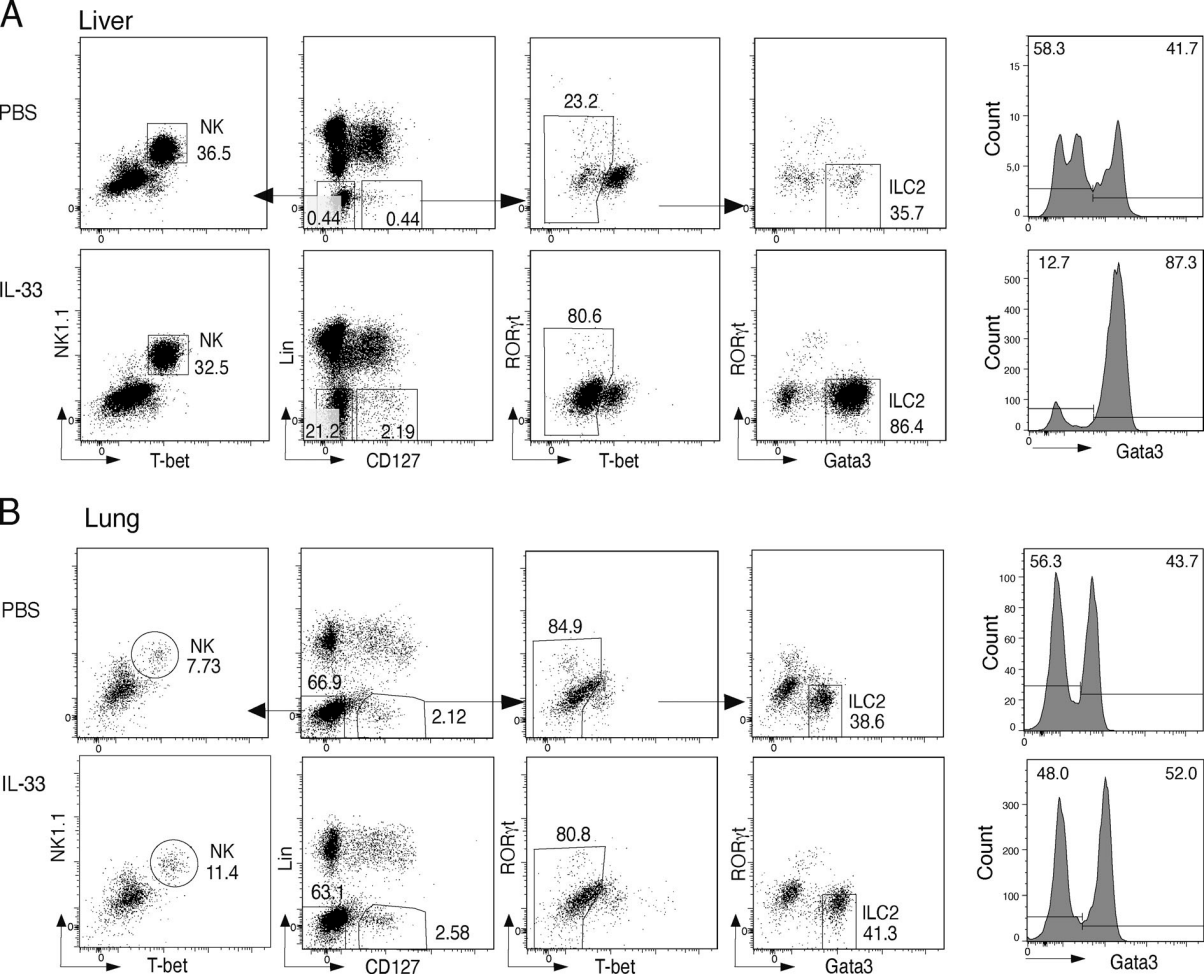
**Sorting strategy for ILC2 and NK cells from lung and liver. (A and B)** ILC2 were sorted as Lin (CD3 and CD19)^−^CD127^+^Tbet^−^Gata3^+^RORγt^−^; NK cells were sorted as Lin^−^CD127^−^NK1.1^+^Tbet^+^ from lung (A) and liver (B) of both control and IL-33–treated mice. The histograms depicted the Gata3 expression against cell count.

**Figure 6.**
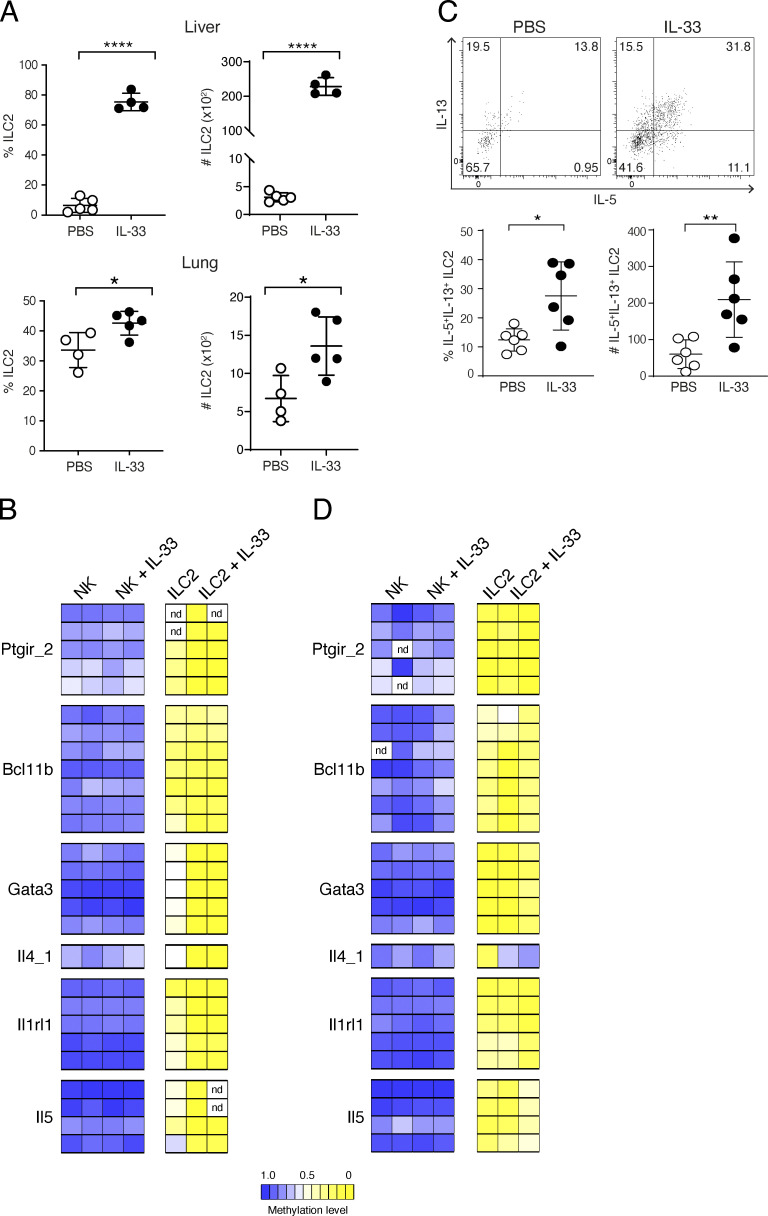
**Impact of IL-33–mediated challenge on ILC2-associated epigenetic marker in lung and liver. (A)** NK cells and ILC2 were isolated from the liver or lung of mice under homeostatic conditions or after i.p. treatment with 300 ng IL-33 for three consecutive days (liver) or i.n. treatment for three consecutive days with 250 ng IL-33 (lung). Graphs show frequencies and total numbers of Gata3^+^ ILC2 within Lin^−^CD127^+^ cells. Data shown were generated from *n* = 4–5 independent cell sorts with *n* = 7–8 mice per sort. Statistical significance was analyzed using unpaired two-tailed Student’s *t* test with *, P ≤ 0.05 and ****, P ≤ 0.0001. **(B)** Pyrosequencing results show the methylation value of selected ILC2-associated marker regions in liver NK cells and ILC2 of both naive and IL-33–treated mice. The mean methylation value of all CpG motifs within each region was calculated and transformed into a color-coded box, ranging from blue (100% methylation) and white (50% methylation) to yellow (0% methylation). Data for untreated mice were generated by pooling ILC2 from several cell sorts with *n* = 20–30 mice per sort. *n* = 2 independent experiments were conducted for IL-33 challenge with *n* = 16 mice. **(C)** ILC2 were isolated from the lung of naive or IL-33–challenged mice and restimulated with PMA*/*ionomycin in vitro for intracellular cytokine staining. Representative flow cytometry plots of Gata3^+^ ILC2 producing IL-5/IL-13 from control (PBS) and IL-33–challenged mice. Graphs show frequencies and total cell numbers of Gata3^+^IL-5^+^IL-13^+^ ILC2. Data shown from *n* = 2 independent experiments with *n* = 3 mice per group. Statistical significance was analyzed using unpaired two-tailed Student’s *t* test with *, P ≤ 0.05 and **, P ≤ 0.01. **(D)** Pyrosequencing results show the methylation value of ILC2-associated marker regions in lung NK cells and ILC2 of both naive and IL-33–treated mice. The mean methylation value of all CpG motifs within each region was calculated and transformed into a color-coded box, ranging from blue (100% methylation) and white (50% methylation) to yellow (0% methylation). Data for untreated mice were generated by pooling ILC2 from several cell sorts with *n* = 10–20 mice per sort. *n* = 2 independent experiments were conducted for IL-33 challenge with *n* = 5 mice.

Tissue location and environmental challenges, such as the presence of inflammatory cytokines, can have a profound effect on the transcriptional signature of ILC2 (Ricardo-Gonzalez et al., 2018). To assess the impact of tissue location and inflammatory stimuli on the methylation level of ILC2-associated epigenetic marker regions, we analyzed ILC2 isolated from liver or lung of either untreated or IL-33–challenged mice (Fig. S5, A and B). In agreement with a recent study (Neumann et al., 2017), we could demonstrate a substantial increase in the frequencies and total numbers of liver ILC2 by IL-33 treatment (Fig. 6 A). However, the cell numbers under homeostatic conditions were very low and enabled just a single analysis of a restricted epigenetic marker panel. Interestingly, we found only intermediate methylation levels of *Gata3* (53%), *Il4* (56%), *Il5* (49%), or *Il1rl1* (36%) regions in ILC2 under homeostasis, while ILC2 isolated under inflammatory conditions displayed clearly reduced methylation of these loci (*Gata3* [3%], *Il4* [3%], *Il5* [7%], and *Il1rl1* [8%]). (Fig. 6 B and Table S4). Notably, NK cells isolated as controls from the same animals did not show demethylation of the ILC2 marker regions under either homeostatic or inflammatory conditions (Fig. 6 B). A small population of ILC2 could be identified in the lungs of untreated mice (Fig. 6 A). However, in contrast to the liver, ILC2 marker regions were already highly demethylated under homeostatic conditions (Fig. 6 D and Table S3). IL-33–mediated challenge increased frequencies and total numbers of lung ILC2 and induced IL-5 and IL-13 secretion by these cells (Fig. 6, A and C), but did not markedly impact the status of ILC2 marker regions, except for *Il4*, where we even saw an increase in methylation (Fig. 6 D). Again, control NK cells sorted from the lung of untreated and IL-33–challenged mice did not show demethylation of ILC2 marker regions (Fig. 6 D). In summary, our data indicate that location affects the methylation status of ILC2 marker regions. Acute IL-33–mediated challenge induced further demethylation of ILC marker regions only in the liver, where ILC2 regions displayed intermediate levels of demethylation during homeostasis.

## Discussion

The study of global epigenomic features and transcriptomic programs provides important clues to the nature of ILC identity and allows novel insights into ILC lineage heterogeneity, plasticity, and function. However, while epigenetic mechanisms have been broadly studied in T cells, only a few studies have related to the epigenetic control of ILCs. These studies mainly focused on histone modification, chromatin accessibility, and long noncoding RNAs (Antignano et al., 2016; Gury-BenAri et al., 2016; Koues et al., 2016; Liu et al., 2017; Mowel et al., 2017; Shih et al., 2016), whereas the impact of DNA methylation has so far not been assessed in ILCs. Using a genome-wide bisulfite sequencing approach, we identified DMRs in murine ILCs and provide data sets at single-CpG resolution. Closer inspection of DMRs and their associated genes allowed us to define and characterize a specific set of marker regions for each ILC lineage.

The global assessment of the WGBS data revealed that the majority of DMRs found in ILCs were mapped to intragenic regions, with a peak of demethylation close to the TSS of genes, mirroring previous observations in T cells (Delacher et al., 2017; Hashimoto et al., 2013; Yang et al., 2015). Total DMR numbers and Euclidian sample distance analysis of the WGBS data also indicated that NK cells share fewer similarities with helper ILCs, which might reflect their functional difference and divergence from the helper ILC populations at the early stage of ILC development (Stokic-Trtica et al., 2020).

Previous work demonstrated accessible chromatin and permissive histone marks within the *Tbx21* locus in ILC1 and the *Gata3* locus in ILC2 (Gury-BenAri et al., 2016; Shih et al., 2016). In line with these studies, we identified specific DMRs in *Tbx21* in ILC1 and in *Gata3* in ILC2. Interestingly, we found that while lineage-specific DMRs in *Tbx21* and *Gata3* were located in introns, the promoter regions of *Tbx21* and *Gata3* were fully demethylated in all ILC subsets. Demethylation of the promoter region of *Gata3* might in part be explained by the fact that Gata3 is expressed by all helper ILCs during their development (Yagi et al., 2014). Nevertheless, low methylation of the promoter may rather indicate a poised status, and thus contribute to lineage plasticity of ILCs and the potential to express more than one lineage-specific transcription factor under inflammatory conditions. Importantly, the identification of lineage-specific DMRs in *Tbx21* and *Gata3* within introns suggests a vital role of intragenic methylation in the regulation and stability of cell type–specific transcription. This is in line with the observation that promoters are preferentially enriched with epigenetic regulatory elements common to all ILC populations but absent from variable and lineage-specific regulatory elements (Shih et al., 2016).

A principle aim of this study was the identification of lineage-specific epigenetic marker regions for helper ILCs. The main criterion defining such marker regions was a high methylation difference value in one-to-one ILC lineage comparisons. This approach allowed us to identify marker regions associated with genes that have not been described for any specific function in ILCs so far, and therefore may represent interesting novel targets for future functional studies. The ILC1 signature contains known marker genes such as *Tbx21*, *Ifng*, and *Il2rb,* whose methylation levels are comparable to those of NK cells. Interestingly, the importance of *Runx2* was described in human but not in murine ILC1 (Allan et al., 2017). The CBP/p300 interaction partner Trerf1 and the chemokine receptor Gpr18, which is involved in recruiting of intraepithelial lymphocytes, were not reported so far. This also holds true for the well-known human NK marker Klrc1 (NKG2A; Crinier et al., 2018). Most ILC3 marker regions show comparable methylation values in LTi cells. This similarity was expected, because both populations express the transcription factor RORγt and were separated in our approach by the expression of CCR6 in LTi cells. The marker region in *Ccr6* shows, besides LTi cells, also intermediate methylation levels in ILC3, but no gene expression under homeostatic conditions, indicating former expression of the receptor. A match in methylation values and gene expression was also discovered for the ILC3 marker *Il7r* and *Blk*, a nonreceptor tyrosine kinase important for γδ T cell development (Malhotra et al., 2013). Although they displayed lower methylation levels in ILC3, we found that LTi cells displayed higher gene expression of Lrrk2, a widely expressed leucine-rich repeat kinase that is probably involved in immune control (Ahmadi Rastegar and Dzamko, 2020); *Pxdc1*, a gene of unknown function; and *Vipr2*, involved in regulatory circuits and tissue protection (Seillet et al., 2020). A unique upregulation of Emb, a transmembrane glycoprotein, was observed in ILC3, whereas Exoc6, the Exocyst complex component 6 that is involved in vesicle transport (Mukerji et al., 2012), was dominantly produced in ILC1. Skewed patterns for ILC3 and LTi can be explained by a still-flexible differentiation level present in second lymphoid organs or by performing the RNA-seq under homeostatic conditions. As already reported by others, the expression pattern might change under inflammatory conditions in the tissue (Zeis et al., 2020).

Based on the nearly perfect match between the epigenetic ILC2 marker regions and our expression data, we decided to use the ILC2 signature for further analysis of the cells. In a first step, we investigated the methylation status in other immune cell subsets, which revealed clear demethylation of the *Bcl11b*-associated marker region also in naive and memory T cell populations, which was not unexpected considering the importance of Bcl11b not only for ILC2, but also in T cell lineage development and maintenance (De Obaldia and Bhandoola, 2015). *Ptgir* en-codes for the prostaglandin I2 (PGI2) receptor, which transmits inhibitory signals to ILC2 (Zhou et al., 2016). PGI2 receptor–mediated downregulation of inflammation has also been described for myeloid cells and T cells, which is in accordance with our finding of increased demethylation of *Ptgir* in those populations (Zhou et al., 2007). Markers associated with *Neb* (Nebulin) and *Ptpn13* (protein tyrosine phosphatase non-receptor type 13), two genes encoding for proteins with unknown function, showed specific demethylation in ILC2. Of note, specific expression of *Neb* and *Ptpn13* was observed not only in LN-derived ILC2s, but also in ILC2 sorted from the small intestine, which may indicate a particular function of these genes in ILC2s (Gury-BenAri et al., 2016). Interestingly, the marker region that we identified in choline dehydrogenase (*Chdh*), encoding for an enzyme in the choline metabolism in mitochondria, is located in close proximity to the promoter of *Il17rb*, the receptor for IL-25. It will be interesting to test whether this epigenetic marker region may equally regulate the expression of both genes. Our data confirm that both Chdh and Il17rb are highly expressed in LN-derived ILC2. In line with that, specific expression of these two genes in ILC2 was also observed previously in the small intestine and lung, and is considered to be part of the tissue-specific profile of ILC2 (Ricardo-Gonzalez et al., 2018; Robinette et al., 2015).

Our DMR-based KEGG pathway analysis indicated an overlap of regulatory processes in CD4^+^ T cells and ILCs. This is probably due to the expression of the same line transcription factors T-bet, Gata3, and RORγt. As the development from naive into memory T cells is accompanied by epigenetic imprinting, we were interested to identify differences and similarities in this regulatory layer between ILCs and Th cells. Surprisingly, the initial analysis of DMRs from pairwise comparisons of all populations did not lead to a clustering of populations that express the same lineage transcription factor, such as NK and ILC1 or ILC3 or LTi (Fig. S1 D), but resulted in a clear separation of ILCs and Th cells (Fig. 4 A). Deeper analysis of the specifically demethylated DMRs between the two sample groups indicated a dominant regulatory network for the T cell receptor and associated proteins in T cells, whereas genes coding for immunoregulatory proteins, which were expressed in a variety of immune cells, were affected in ILCs. Future comparisons of the transcription factor–driven network will show which pathways are unique in ILCs and Th cells.

A more detailed analysis of the ILC2 signature revealed a similar methylation pattern between ILC2 and Th2 cells in *Ptgir*, *Chdh*, *Bcl11b*, *Il4*, *Il5*, and *I1rl1*. The demethylation in *Nmur1*, *Gata3*, and *Neb* was less pronounced in Th2 cells, and no clear similarity was seen in *Rem2* and *Ptpn13*. To clarify whether different regulatory elements bind mostly non–T cell transcription factors, we searched for overrepresented regions and corresponding transcription factor binding sites within ILC2-specific DMRs. After filtering for those transcription factors expressed in ILC2, we surprisingly identified primarily protein families such as TCF/LEF, IRF, POK/ZBTB, STAT, JUN, and FOS, known to be responsible for development and function of T cells (Evans and Jenner, 2013; Kang and Malhotra, 2015). These results suggest that similar sets of factors may play a role during ILC and T cell development. Detailed studies must show whether gene loci that play a role in both populations are regulated by the same or different transcription factor complexes. Interestingly, the unequal methylation profile of *Gata3* in ILC2 and Th2 cells indicates recruitment of transcription factor complexes to different sites.

For Th cells, several studies have suggested that environmental signals may critically shape their epigenetic signatures. For example, differences in the degree of methylation were observed in ex vivo Th17 vs. in vitro differentiated Th17 cells (Yang et al., 2015), or between ex vivo Tregs and in vitro differentiated Tregs (Floess et al., 2007). Recently, a genome-wide analysis also revealed differences in the DNA methylation of Tregs derived from different tissues (Delacher et al., 2017). Here, we analyzed the methylation status of ILC2-associated marker regions in ILC2 from lung and liver. Under homeostatic conditions, NK cells and ILC1 constituted the main ILC populations in the liver, whereas ILC2 were found to be very rare. In contrast, ILC2 represent the main ILC lineage in the lung (Dutton et al., 2017). Although we were able to analyze only a restricted amount of marker in the liver, because of the very low number of ILC2 that could be isolated from this organ during steady state, our results indicate overall higher methylation levels compared with lung- or LN-derived ILC2s. Whether this reflects functional differences or an earlier state of maturation is not clear, but nevertheless suggests that distinct local conditions can impact on the epigenetic status of the cells. Interestingly, if there is an influx of mobilized ILCs into the liver, those are true ILC2 as confirmed by the demethylated signature.

Analyzing the impact of an acute inflammatory stimulus on the methylation status of ILC2 marker regions in lung ILC2 revealed only minor changes, except for *Il4*. We even saw a clear methylation increase for *Il4* and a similar, slight trend for the rest of the ILC2 signature. This might reflect the different response of heterogeneous ILC2 subpopulations in the lung, with a major proliferative boost in not completely maturated ILC2, as observed during a *Nippostrongylus brasiliensis* infection (Zeis et al., 2020). The IL-33 model, characterized by IL-5 and IL-13 expression, seems to lack triggers for IL-4 upregulation and might result in methylation increase of the *Il4* gene locus. In view of the fact that the IL-33–mediated challenge clearly enhanced demethylation of ILC2 marker regions in the liver, we hypothesize that environmental challenges can impact the methylation status, depending on the tissue that is affected. In addition, the kind and strength of the inflammatory stimuli may influence on the expansion of ILCs and the dynamics of methylation patterns. Therefore, we cannot rule out that the epigenetic signature might slightly vary at different time points in the lung.

Despite the general classification of ILCs into main lineages, research during the last years has revealed a substantial degree of phenotypical and functional heterogeneity within ILC subsets. Recent single cell omics approaches moreover suggest significant inter- and intralineage plasticity and describe multiple transitional states both under homeostatic conditions and ongoing immune responses (Bielecki et al., 2021; Zeis et al., 2020). Importantly, we defined the populations for our initial WGBS by the expression of one of the master transcription factors T-bet, Gata3, and RORγt, and excluded the expression of the other two transcription markers. This way, we reduced the heterogeneity of our populations and the likelihood of missing specific DMRs due to the presence of overlapping subpopulations. Nevertheless, the sorted populations still represent a mixed population, as seen, for example, by the heterogeneous expression of IL17RB and ST2 in mesenteric vs. inguinal ILC2. As a consequence of this approach, the regions that we identified may rather represent a core signature that can be used to study ILCs from different tissues or under specific immunological conditions, as we did in our lung and liver inflammation models. Since we defined epigenetic marker regions by comparing ILCs from pLNs, we most likely were not able to identify markers associated with specific location or function in tissues such as the skin, lung, or intestine. In addition, as we sorted the cells for our initial WGBS analysis from adult mice under steady-state conditions, it is possible that we missed specific markers associated with early development, activation, or innate memory function.

In summary, we believe that our genome-wide approach to identify specific regions of CpG demethylation in ILCs will help to better define ILC identities, facilitate their identification, and help to better understand the molecular programs that underlie their function at different tissue locations.

## Materials and methods

### Mice

Populations of ILCs for WGBS and pyrosequencing analyses were sorted from 8–20-wk-old male and female C57BL/6J mice. Th cells for WGBS were sorted from 34–38-wk-old *RORc(gt)-Gfp*^*TG*^-*Foxp3-IRES-mRFP* mice (Yang et al., 2016a). Adult 8–18-wk-old *RORc(gt)-Gfp*^TG^ transgenic reporter mice (Lochner et al., 2008) were used for RNA-seq*.* Adult 12–16-wk-old *Rosa26-Cas9*^GFP^ mice (Chu et al., 2016) were used for CRISPR/Cas9-mediated gene targeting*.* All mice were bred on a C57BL/6J background. All mice were bred and maintained under specific pathogen–free conditions in animal facilities of TWINCORE (Hannover, Germany), Helmholtz Centre for Infection Research (Braunschweig, Germany), or University Medical Centre Hamburg Eppendorf (Hamburg, Germany). Animal experiments were performed under approval by the Lower Saxony Committee on the Ethics of Animal Experiments as well as the responsible state office (Lower Saxony State Office of Consumer Protection and Food Safety) under permit number 33.9-42502-04-19/3284 (intranasal IL-33 treatments). IL-33–induced liver inflammation was approved by the institutional review board (G44/15 and G58/17; Behörde für Gesundheit und Verbraucher-schutz, Hamburg, Germany). All animal experiments were carried out in accordance with the German Animal Welfare Act, the German Animal Welfare Ordinance, and the Directive of the European Parliament and Council for the protection of animals used for scientific purposes (2010/63/EU).

### Antibodies

LIVE/DEAD Fixable Dead Cell Stain Kit was purchased from Life Technologies/Thermo Fisher Scientific. The following antibodies were purchased from eBioscience/Thermo Fisher Scientific: APC-conjugated anti-mouse CD3 (145-2C11), CD19 (1D3), TCRβ (B20.1), TCRγδ (eBioGL3), CD11b (M1/70), CD11c (N418), NK1.1 (PK136), Gr-1(RB6-8C5), CD4 (GK1.5), CD14 (Sa2-8), and CD44 (IM7); FITC-conjugated anti-mouse NK1.1 (PK136) and CD11b (M1/70); PE-conjugated anti-mouse CD127 (A7R34), CD3 (145-2C11), and IL-13 (eBio13A); PerCP/eF710-conjugated anti-mouse/human Gata3 (TWAJ) and CD62L (MEL-14); PerCP/Cy5.5-conjugated anti-mouse CD4 (RM4-5), eF450-conjugated anti-mouse IL-5 (TRFK5); AF488-conjugated anti-mouse Foxp3 (FJK-16 s), PerCP/Cy5.5-conjugated anti-mouse Gata3 (TWAJ), and PE-conjugated anti-mouse T-bet (4B10). The following antibodies were purchased from BioLegend: PE-conjugated anti-mouse IL-17RB (9B10), BV605 conjugated anti-mouse CD127 (A7R34), BV605-conjugated anti-mouse CCR6 (29-2L17), PE/Cy7-conjugated anti-mouse/human T-bet (4B10), PE/Cy7-conjugated anti-mouse ST2 (DIH9), BV421-conjugated anti-mouse/human KLRG1 (2F1/KLRG1), APC/Fire 750-conjugated anti-mouse NKp46 (29A1.4), APC/Cy7-conjugated anti-mouse CD3ε (145-2X11), PE/Cy7-conjugated anti-mouse/human CD44 (IM7), and BV605-conjugated anti-mouse CD62L (MEL-14). BV421-conju-gated anti-mouse RORγt (Q31-378) and V500-conjugated anti-mouse CD4 (RM4-5) were purchased from BD Bioscience.

### Flow cytometry

Cells were washed with PBS twice and resuspended with PBS at a concentration of 5 × 10^7^ cells in 1,250 μl PBS. 1 μl of LIVE/DEAD Fixable Dead Cell Stain Kit (Invitrogen) was added into cells per 500 μl final staining volume to exclude dead cells. After incubating at 4°C for 20 min, cells were washed twice with PBS containing 2% FCS. After washing, CCR6 was labeled at 37°C for 20 min before labeling any other surface marker. Afterward, cells were labeled with lineage markers in a final concentration of 5 × 10^7^ cells in 1,250 μl FACS buffer at 4°C for 30 min. For intracellular staining, cells were fixed and permeabilized with Foxp3/Transcription Factor Staining Buffer Set (eBioscience) according to the manufacturer’s instructions, followed by labeling with intracellular transcription factors overnight. For cytokine staining, cells (≤1 × 10^6^) were first incubated in 200 µl RPMI-1640 Glutamax (Gibco) containing 10% FCS (Biochrom), 10 mM Hepes (Gibco), 50 µM β-mercaptoethanol (Gibco), 100 ng/ml PMA (Sigma-Aldrich), 1 μg/ml ionomycin (Sigma-Aldrich), and 5 μg/ml Brefeldin A (eBioscience) at 37°C for 3.5 h. FACSAria II (BD Bioscience) and LSR II (BD Bioscience) were used for sorting or acquiring samples. Sorting was conducted at the Flow Cytometry and Cell Sorting platform (Helmholtz Center for Infection Research, Braunschweig) or Central Research Facility Cell Sorting (Hannover Medical School). Data were analyzed with FlowJo software (TreeStar).

### IL-33–mediated lung and liver challenge models

To induce lung inflammation, mice were treated daily on three consecutive days with 250 ng recombinant murine IL-33 (PeproTech) i.n. To induce liver inflammation, mice were treated daily on three consecutive days with 300 ng rmIL-33 (Bio-Legend) i.p. Control mice were treated with PBS (Life Technologies). 24 h after the last treatment, mice were sacrificed, and lungs or livers were processed to obtain single-cell suspensions for sorting ILC2 and NK cells. ILC2 were sorted as Lin (CD3 and CD19)^−^CD127^+^Tbet^−^Gata3^+^RORγt^−^; NK cells were sorted as Lin (CD3 and CD19)^−^CD127^−^NK1.1^+^Tbet^+^.

### Preparation of single-cell suspensions from murine tissues

Spleens and pLN were extruded through 70-μm cell strainers into 50-ml Falcon tubes by syringe plungers. After rinsing the strainers with PBS containing 2% FCS, cells were centrifuged. Cell pellets were resuspended with 2 ml RBC lysis buffer and incubated at room temperature for 2 min. 8 ml PBS containing 2% FCS were added to stop the lysis. Cells were washed and resuspended with PBS containing 2% FCS at a concentration of 4 × 10^6^ cells/ml for further experiments.

Lung was first washed with PBS and dried on a clean tissue paper to remove remaining blood. After being cut into very fine pieces, the lung was digested with 4 ml RPMI-1640 Glutamax containing 5% FCS, 10 mM Hepes, 1 mg/ml Collagenase D (Roche), and 0.1 mg/ml DNase (Roche) at 37°C for 1 h. Afterward, the remaining lung pieces and digestion solution were extruded through 100-μm cell strainers into 50-ml Falcon tubes by syringe plungers and filled up to 20 ml with PBS containing 2% FCS. Following centrifugation, 14.7% Optiprep (Axis-Shield) gradient was conducted to obtain mononuclear cells. After the gradient, the mononuclear cell layer was collected into PBS containing 2% FCS for further experiments.

Liver was harvested after performing systemic perfusion by injecting 10 ml cold PBS twice from the left ventricle to the right atrium. The gall bladder was carefully removed, and the harvested liver was cut into fine pieces. The tissue pieces were further digested with 4 ml RPMI-1640 Glutamax containing 5% FCS, 10 mM Hepes, 1 mg/ml Collagenase D, and 0.1 mg/ml DNase at 37°C for 45 min. The remaining liver pieces and digestion solution were extruded through 100-μm cell strainers into 50-ml Falcon tubes by syringe plungers and filled up to 50 ml with PBS containing 2% FCS. Cells were then centrifuged without break at 100 *g*, room temperature, and highest acceleration speed for 1 min. Supernatant was collected and centrifuged again at 800 *g* and room temperature for 10 min to obtain compact cell pellets. 40–80% Percoll (GE Healthcare) gradient was applied to obtain a mononuclear layer. The mononuclear cell layer after the gradient was collected into PBS containing 2% FCS for further experiments.

### CRISPR/Cas9-mediated gene targeting of ILC2

ILC2 were sorted as Lin^−^Cas9^GFP+^CD127^+^NKp46^−^KLRG1^+^ from pLNs of *Rosa26-Cas9*^*GFP*^ mice. Lineage markers included CD3, CD19, TCRαβ, TCRγδ, CD11c, CD11b, Gr-1, CD4, CD14, and NK1.1. Sorted ILC2 (≤10.000 cells) were cultured in 96-well U plates with 200 μl culture medium at 37°C for 8 d. The culture medium consisted of RPMI-1640 Glutamax containing 10% FCS, 10 mM Hepes, 50 µM β-mercaptoethanol, 100 μg/ml Primocin (InvivoGen), 20 ng/ml rmIL-7 (PeproTech), 20 ng/ml rmIL-33 (PeproTech), and 25 U IL-2 (NIH). The medium was changed every 3 d. Control Th2 cells were differentiated from naive CD4^+^ T cells as described previously (Berod et al., 2014). Cultured ILC2 were washed and resuspended with OptiMEM at the concentration of 1 × 10^6^ cells/ml before electroporation. sgRNAs were complexed by incubating 2.5 μl CRISPR RNA (crRNA; 200 μM) and 2.5 μl tracrRNA-ATTO550 (200 μM) together at 95°C for 6 min. After the incubation, the sgRNAs were removed from heat and cooled to room temperature. 45 μl OptiMEM, 5 μl sgRNA complex, and 50 μl cells were mixed and transferred into a 2-mm Gene Pulser Cuvette (Bio-Rad). Electroporation was conducted on the Gene Pulser X (Bio-Rad) at 280 V, square wave, 1 pulse, no interval time for 2 ms. After electroporation, cells were cultivated in the culture medium without cytokines at 37°C for 2 h. After resting, cells were washed and cultured with the culture medium described above containing 20% FCS at 37°C for 3 d. crRNA recognizing *Nmur1*-associated marker region was 5′-GTG​AAG​AGA​AAG​CCA​CCG​TAG​GG-3′. Negative control crRNA, tracrRNA-ATTO550, and *Nmur1*-specific crRNA were purchased from Integrated DNA Technologies.

To test the effect of *Nmur1*-targeting on gene expression, RNA was obtained from ILC2 on day 3 after electroporation using AllPrep DNA/RNA Mini Kit (Qiagen). RNA was transcribed into cDNA using SuperScript III Reverse Transcriptase Kit (Thermo Fisher Scientific). Quantitative PCR was performed using iQ SYBR Green Supermix (Bio-Rad) on a LightCycler 480 II (Roche). All procedures were performed according to the manufacturers’ instructions. Gene expression was normalized to *Actb* and log2 transformed. Primers for *Actb* were obtained from Eurofins MWG: Actb (forward, 5′-TGT​TAC​CAA​CTG​GGA​CGA​CA-3′; reverse, 5′-GGG​GTG​TTG​AAG​GTC​TCA​AA-3′). PrimePCR Nmur1 Template (Bio-Rad) was used to analyze the expression of *Nmur1*.

### WGBS

Fixed Lin^−^ (CD3^−^CD19^−^) cells were sorted at high purity for NK cells (CD127^−^NK1.1^+^T-bet^+^), ILC1 (CD127^+^RORγt^−^Gata3^−^T-bet^+^), ILC2 (CD127^+^RORγt^−^Gata3^+^T-bet^−^), ILC3 (CD127^+^RORγt^+^Gata3^−^T-bet^−^CCR6^−^), and LTi cells (CD127^+^RORγt^+^Gata3^−^T-bet^−^CCR6^+^). Several rounds of sorts were conducted to obtain ≥220,000 cells for each ILC population. CD4^+^ cells from spleen and lymph nodes were magnetically enriched by using anti-CD4 microbeads and the autoMacs Pro separator (Miltenyi Biotec). The enriched CD4^+^ cells were fixed and sorted in high purity for Th1 (CD3^+^CD4^+^Foxp3^−^CD44^hi^CD62L^low^T-bet^+^), Th2 (CD3^+^CD4^+^Foxp3^−^CD44^hi^CD62L^low^Gata3^+^), and Th17 cells (CD3^+^CD4^+^Foxp3^−^CD44^hi^CD62L^low^RORγt^+^). Genomic DNA from the sorted cells was extracted using the NucleoSpin Tissue kit (Macherey-Nagel), including a crosslink removal step that was described recently (Kyburz et al., 2019). Briefly, Chelex-100 beads (Bio-Rad) were added after the lysis step and incubated at 95°C for 15 min in a shaker. Chelex-100 beads were spun down, and the supernatant was transferred to a fresh tube. The resulting single-stranded DNA was converted with sodium bisulfite using the EZ DNA Methylation-Direct Kit (Zymo Research) and fragmented by sonication (Covaris S220, 10% duty cycle, 175 W peak incident power, intensity 5, 200 cycles per burst, 120 s). The fragmented DNA served as input for the Accel-NGS Methyl-Seq DNA Library Kit (Swift Biosciences) and resulted in libraries that were sequenced on an Illumina NovaSeq 6000 with depths of 210 million to 282 million paired-end reads (2 × 150 bp). The sequenced libraries were assessed for sufficient sequencing quality and potential adapter contamination by using the programs FastQC (Babraham Bioinformatics; https://www.bio-informatics.babraham.ac.uk/projects/fastqc/), trim_galore (Babraham Bioinformatics; http://www.bioinformatics.babraham.ac.uk/projects/trim_galore/), and cutadapt (Martin, 2011). Sequencing reads (R1/R2) were trimmed by adapter and low-quality-end (Phred score <20) removal, followed by deletion of 10 bases at 5′ and 3′ ends to avoid a bias by insufficient end-repair reaction. Reads that were shorter than 20 nucleotides after trimming were discarded. Quality-controlled libraries have been mapped against the mouse reference genome (GRCm38, without gonosomes) using the bisulfite short read mapping software BSMAP (Xi and Li, 2009). To accurately measure the methylation levels of cytosines within CpG motifs and obtain corresponding coverage information, methylation level calling was taken into account only for properly paired unique read pair mappings (methratio.py parameters: --unique, --paired, --remove-duplicate). Because of the high sequencing depth, we were able to assay the methylation level of 19,987,113 CpGs, comprising >98% of all 20,383,910 CpGs in the mouse genome at least one time. Moreover, >76% of all CpGs have been assayed at least five times (i.e., read coverage ≥5) in all samples. The reliable detection of DMRs even in the absence of replicates was performed with the software tool metilene (Juhling et al., 2016). We considered for the detection process only CpG motifs with a coverage of at least five that are present in at least one sample, and a DMR had to contain at least three CpGs. Further on, to be classified as DMR, the difference in mean methylation (i.e., mean over all CpGs of a region) of a region between two compared samples/conditions had to be ≥25%. The resulting DMRs have been further classified according to their genomic location into classes (a) promoter, (b) intragenic, or (c) intergenic using mouse annotations from Ensembl release 81. The promoter region was defined as 1 kb around the TSS. TSS coordinates of annotated transcripts have been used further to compute the histogram of distances between DMRs and nearest TSSs. DMRs located downstream of the TSS distances are positive, whereas DMRs upstream of the TSSs have negative distances. The distances of DMRs overlapping the TSS are counted as zero.

To further investigate the main differences among the methylomes of ILCs (ILC1, ILC2, ILC3, LTi, and NK cells) and CD4^+^ Th cells (Th1, Th2, and Th17 cells), we include all unique DMRs from ILC vs. Th cell pairwise comparison with a mean methylation difference of ≥0.5 in all comparisons that are located in a 5-kb proximity around the TSS of an annotated gene. DMRs located close to genes without canonical gene names were excluded. The DMR methylation values from all samples (*n* = 8) were plotted for each of the top 75 hypermethylated and top 75 hypomethylated DMRs grouped by their nearest gene (Fig. 4, B and C). If more than one DMR (*k* ≥ 1) was identified in a gene locus, k*n data points were plotted.

### Smoothing of methylation values for visualization

The raw methylation values of the samples were smoothed by BSmooth (Hansen et al., 2012) as implemented in the R/Bioconductor package bsseq and used to compute the Euclidian distances for hierarchical clustering and heatmap visualization. Graphic output was generated by the R/Bioconductor package GViz (Hahne and Ivanek, 2016). Information on the genomic location of the regions shown in the methylation profiles (Figs. 2 B and 3 D) are provided in Table S1 B.

### Motif discovery and identification

For de novo discovery of conserved sequence motifs in DMRs, we used an expectation maximization approach as implemented in MEME software (Bailey and Elkan, 1994). Therefore, we extended the DMRs detected in comparisons ILC2 vs. ILC1, ILC2 vs. ILC3, and ILC2 vs. LTi by 10 nucleotides on both ends and performed motif discovery on both strands with MEME (parameters: *-dna -revcomp -nmotifs 20 -mod anr*). Low-complexity motifs [e.g., poly(A/T)] were removed from the resulting lists, and motifs with an E value <0.05 were subjected to motif identification using TOMTOM software (Gupta et al., 2007) and the HOCOMOCO database (v11, core_mouse) of mouse transcription factor binding models (Kulakovskiy et al., 2018).

### Pathway enrichment analysis

To detect pathways that might be modulated by methylation changes, we performed a pathway enrichment analysis on the set of genes associated with a DMR. For this, we associated in a first step each DMR (mean methylation difference ≥0.25, P value ≤0.01) from one pairwise comparison with its nearest annotated gene and looked up all KEGG metabolic pathway annotations (Kanehisa et al., 2016) of those genes. In a second step, we used hypergeometric testing as implemented in the R package gostats (Falcon and Gentleman, 2007) on the list of annotated KEGG pathways to determine significantly (P value ≤0.01) overrepresented pathways. From the resulting pathway list, we took the 40 pathways with highest odds ratios that are overrepresented in all pairwise comparisons and generated a pseudo-heatmap using the color-coded odds ratio values.

### RNA-seq

Lymph node cells from *RORc(gt)-Gfp*^TG^ reporter mice were sorted as previously described (Gury-BenAri et al., 2016). Briefly, ILCs were identified using the lineage marker panel and antibodies against CD127, CCR6, KLRG1, and NKp46 to sort triplicates of ILC1 (Lin^−^RORγt^Gfp−^CD127^+^KLRG1^−^NKp46^+^), ILC2 (Lin^−^RORγt^Gfp−^CD127^+^KLRG1^+^NKp46^−^), ILC3 (Lin^−^RORγt^Gfp+^CD127^+^CCR6^−^), and LTi cells (Lin^−^RORγt^Gfp+^CD127^+^CCR6^+^). RNA was purified from the sorted cells using the RNeasy Micro Kit (Qiagen) according to the manufacturer’s protocol, subjected to cDNA synthesis, and amplified using template-switching technology of the SMART Seq v4 Ultra Low Input RNA Kit (Takara Bio), followed by purification using the Agencourt AMPure XP Kit (Beckman Coulter). Library preparation was performed with Nextera XT DNA Library Prep Kit (Illumina). Sequencing was carried out on an Illumina HiSeq 2500 (50-bp single-end reads) resulting in a sequencing depth of 25.5 million to 39.2 million reads per sample. All libraries were assessed for sufficient read quality (Phred score >30) and potential contamination using the FastQC program. Identified adapter contamination was removed using the programs trim_galore and cutadapt. Trimmed reads with a remaining length of <30 nucleotides were discarded. Libraries were aligned to the mouse reference genome (GRCm38) using the RNA-seq aligner STAR (Dobin et al., 2013) resulting in mapping rates between 93.9 and 98.0%. Reads aligned to annotated genes were quantified with the HTseq-count (https://htseq.readthedocs.io/en/master/) using gene annotations from Ensembl release 81 and served as input for DESeq2 (Love et al., 2014) for pairwise detection and quantification of differential gene expression. For DESeq2 parametrization, we used a β prior and disabled the Cook distance cutoff filtering. All other parameters remained unchanged. For visualization purposes, *rlog* and *vst* count transformations were computed. In addition, reads per kilobase maximum transcript length per million mapped reads values were computed for each library from the raw gene counts. The list of DESeq2 determined differentially expressed genes was filtered with a conservative absolute log_2_ fold-change cutoff of ≥2 and a cutoff for a multiple testing corrected P value ≤0.05.

### Correlation between methylation status and gene expression

To investigate potential correlations between methylation and expression changes, we computed the difference in mean methylation of DMRs associated with certain genes and the change in gene expression (measured on a log_2_ scale) and computed the Pearson correlation coefficient (*R*^2^) between these values. The correlation was classified as negligible (0 ≤ *R* ≤ −0.3), low (−0.3 < *R* ≤ −0.5), moderate (−0.5 < *R* ≤ −0.7), high (−0.7 < *R* ≤ −0.9), or very high (−0.9 < *R* ≤ −1; Hinkle et al., 2002).

### Pyrosequencing

The methylation status of the marker regions in main immune cells and ILC from IL-33–treated and untreated mice was analyzed by pyrosequencing. Genomic DNA from sorted cells was bisulfite converted by using the EZ DNA Methylation-Lightning Kit (Zymo Research) according to the manufacturer’s instructions. Pyrosequencing was performed as described previously (Yang et al., 2016a). Primers for amplification of the region of interest, the corresponding sequencing primers, and the chromosomal positions are listed in Table S1 C.

### Statistical analysis

Real-time PCR and flow cytometry data were tested using unpaired two-tailed Student’s *t* test (GraphPad Prism software v8.4). P values were considered significant as follows: *, P < 0.05; **, P < 0.01; ***, P < 0.001; and ****, P < 0.0001.

### Online supplemental material

Fig. S1 shows the characterization of ILC2 in different lymph nodes, the sorting strategy for ILC WGBS, and hierarchical cluster analysis of ILC DMRs. Fig. S2 depicts additional methylation profiles of CpG motifs within gene loci associated with ILCs. Fig. S3 shows sorting strategy for RNA-seq of LN ILCs, principal component analysis, and correlation analysis between expression and methylation. Fig. S4 demonstrates the sorting of Th cell subsets and main immune cells and the methylation status of ILC1, ILC3, and LTi marker regions in Th1, Th2, and Th17 cells. Fig. S5 depicts the sorting strategy for ILC2 and NK cells from lung and liver. Table S1 shows the localization of ILC marker regions and associated gene locus, localization of methylation profiles shown in Figs. 2 B and 3 D, and amplification and sequencing primer for the ILC2 marker regions analyzed by pyrosequencing. Table S2 shows the 1,000 top DMRs among Th and ILC subsets from unsupervised hierarchical clustering. Table S3 shows the methylation values of single CpG motifs located in ILC2 marker regions of immune cell subsets. Table S4 shows the methylation values of single CpG motifs located in ILC2 marker regions of cells from untreated or IL-33–treated mice.

## Data availability

Sequencing data reported in this paper was uploaded to GEO: accession nos. GSE168209 (ILC) or GSE200507 (Th1, Th2, Th17 cells) for WGBS data and GSE168208 for RNA-seq data.

## Supplementary Material

Table S1 shows localization of ILC marker regions and associated gene locus, localization of methylation profiles shown in Figs. 2 B and 3 D, and amplification and sequencing primer for the ILC2 marker regions analyzed by pyrosequencing.

Table S2 shows the 1,000 top DMRs among Th and ILC subsets from unsupervised hierarchical clustering.

Table S3 shows the methylation values of single CpG motifs located in ILC2 marker regions of immune cell subsets.

Table S4 shows the methylation values of single CpG motifs located in ILC2 marker regions of cells from untreated and IL-33–treated mice.
